# Blood Biomarkers for Detection of Brain Injury in COVID-19 Patients

**DOI:** 10.1089/neu.2020.7332

**Published:** 2020-12-14

**Authors:** Steven T. DeKosky, Patrick M. Kochanek, Alex B. Valadka, Robert S.B. Clark, Sherry H.-Y. Chou, Alicia K. Au, Christopher Horvat, Ruchira M. Jha, Rebekah Mannix, Stephen R. Wisniewski, Max Wintermark, Susan E. Rowell, Robert D. Welch, Lawrence Lewis, Stacey House, Rudolph E. Tanzi, Darci R. Smith, Amy Y. Vittor, Nancy D. Denslow, Michael D. Davis, Olena Y. Glushakova, Ronald L. Hayes

**Affiliations:** ^1^ McKnight Brain Institute, University of Florida College of Medicine, Gainesville, Florida, USA.; ^2^ Department of Critical Care Medicine, Department of Anesthesiology, Pediatrics, Bioengineering, and Clinical and Translational Science, Safar Center for Resuscitation Research, University of Pittsburgh School of Medicine, UPMC Children's Hospital of Pittsburgh, Pittsburgh, Pennsylvania, USA.; ^3^ Department of Neurosurgery, Virginia Commonwealth University, Richmond, Virginia, USA.; ^4^ Department of Critical Care Medicine, University of Pittsburgh School of Medicine, Pittsburgh, Pennsylvania, USA.; ^5^ Department of Critical Care Medicine, Neurology, and Neurosurgery, University of Pittsburgh, Pittsburgh, Pennsylvania, USA.; ^6^ University of Pittsburgh, UPMC Children's Hospital of Pittsburgh, Pittsburgh, Pennsylvania, USA.; ^7^ Department of Critical Care Medicine, University of Pittsburgh School of Medicine, Division of Pediatric Critical Care, UPMC Children's Hospital of Pittsburgh, Pittsburgh, Pennsylvania, USA.; ^8^ Departments of Critical Care Medicine, Neurology, Neurological Surgery, Clinical and Translational Science Institute, Safar Center for Resuscitation Research, University of Pittsburgh, Pittsburgh, Pennsylvania, USA.; ^9^ Department of Pediatrics and Emergency Medicine, Harvard Medical School, Department of Medicine, Division of Emergency Medicine, Boston Children's Hospital, Boston, Massachusetts, USA.; ^10^ University of Pittsburgh, Pittsburgh, Pennsylvania, USA.; ^11^ Department of Neuroradiology, Stanford University, Stanford, California, USA.; ^12^ Duke University School of Medicine, Durham, North Carolina, USA.; ^13^ Department of Emergency Medicine, Wayne State University School of Medicine, Detroit Receiving Hospital/University Health Center, Detroit, Michigan, USA.; ^14^ Department of Emergency Medicine, Washington University in St. Louis, St. Louis, Missouri, USA.; ^15^ Department of Emergency Medicine, Washington University School of Medicine, St. Louis, Missouri, USA.; ^16^ Genetics and Aging Research Unit, Massachusetts General Hospital, McCance Center for Brain Health, Massachusetts General Hospital, MassGeneral Institute for Neurodegenerative Diseases, Massachusetts General Hospital, Department of Neurology (Research), Massachusetts General Hospital, Department of Neurology, Harvard Medical School, Charlestown, Massachusetts, USA.; ^17^ Immunodiagnostics Department, Naval Medical Research Center, Biological Defense Research Directorate, Fort Detrick, Maryland, USA.; ^18^ Division of Infectious Disease and Global Medicine, University of Florida, Emerging Pathogens Institute, Gainesville, Florida, USA.; ^19^ Departments of Physiological Sciences and Biochemistry and Molecular Biology, University of Florida, Center for Environmental and Human Toxicology, Gainesville, Florida.; ^20^ Department of Pediatrics, Wells Center for Pediatric Research/Pulmonology, Allergy, and Sleep Medicine, Riley Hospital for Children at Indiana University, Indianapolis, Indiana, USA.; ^21^ University of Virginia Cancer Center, Charlottesville, Virginia, USA.; ^22^ Banyan Biomarkers Inc., Alachua, Florida, USA.

**Keywords:** blood biomarkers, CNS injury, COVID-19, GFAP, SARS-CoV-2, UCH-L1

## Abstract

The severe acute respiratory syndrome coronavirus 2 (SARS-CoV-2) virus attacks multiple organs of coronavirus disease 2019 (COVID-19) patients, including the brain. There are worldwide descriptions of neurological deficits in COVID-19 patients. Central nervous system (CNS) symptoms can be present early in the course of the disease. As many as 55% of hospitalized COVID-19 patients have been reported to have neurological disturbances three months after infection by SARS-CoV-2. The mutability of the SARS-COV-2 virus and its potential to directly affect the CNS highlight the urgency of developing technology to diagnose, manage, and treat brain injury in COVID-19 patients. The pathobiology of CNS infection by SARS-CoV-2 and the associated neurological sequelae of this infection remain poorly understood. In this review, we outline the rationale for the use of blood biomarkers (BBs) for diagnosis of brain injury in COVID-19 patients, the research needed to incorporate their use into clinical practice, and the improvements in patient management and outcomes that can result. BBs of brain injury could potentially provide tools for detection of brain injury in COVID-19 patients. Elevations of BBs have been reported in cerebrospinal fluid (CSF) and blood of COVID-19 patients. BB proteins have been analyzed in CSF to detect CNS involvement in patients with infectious diseases, including human immunodeficiency virus and tuberculous meningitis. BBs are approved by the U.S. Food and Drug Administration for diagnosis of mild versus moderate traumatic brain injury and have identified brain injury after stroke, cardiac arrest, hypoxia, and epilepsy. BBs, integrated with other diagnostic tools, could enhance understanding of viral mechanisms of brain injury, predict severity of neurological deficits, guide triage of patients and assignment to appropriate medical pathways, and assess efficacy of therapeutic interventions in COVID-19 patients.

## Introduction

Infection by the severe acute respiratory syndrome coronavirus 2 (SARS-CoV-2) coronavirus results in significant mortality and long-term disability. Global costs of coronavirus disease 2019 (COVID-19) are predicted to reach as much $35.3 trillion through 2025.^1^ The full spectrum of disease associated with COVID-19 is not yet fully characterized, yet 35% of adult COVID-19 patients report they have not returned to their usual state of health 2–3 weeks after testing positive for the SARS-CoV-2 virus.^2^ Emerging data indicate the presence of brain injury in a subset of COVID-19 patients, consistent with the known ability of coronaviruses to infect the CNS. However, the clinical manifestations, frequency of CNS effects, and associated primary or secondary mechanisms underlying neurological injury produced by SARS-CoV-2 infection are not well understood.

Human coronaviruses have been described as underestimated opportunistic pathogens of the CNS.^3^ The SARS-CoV-2 virus, like many viruses including coronaviruses related to SARS-CoV-2, attacks multiple organs of COVID-19 patients, including the brain. There have been worldwide reports of neurological deficits in COVID-19 patients involving both the central and peripheral nervous systems. However, the pathobiology of CNS infection by SARS-CoV-2 and the associated neurological sequelae of this infection remain poorly understood. A central question is whether or not brain damage in individual patients results from a direct primary effect of the virus on the brain or results indirectly from systemic secondary insults, including hypoxemia, thrombosis, or autoimmune responses. In this review, we outline the rationale for the use of BBs for diagnosis of brain injury in COVID-19 patients, the research needed to incorporate their use into clinical practice, and the improvements in patient management and outcome that can result.

BBs have been successfully used in other acute and chronic brain diseases. Timely implementation of BBs in the current SARS-CoV-2 pandemic will also allow this emerging technology to provide critically needed insights into the risk factors associated with SARS-CoV-2 neurological involvement and the potential for increased risks for long-term neurological deficits and neurodegenerative diseases.

Injury to the peripheral nervous system (PNS) has also been reported in COVID-19 patients.^4–7^ However, in contrast to studies of biomarkers of CNS injury, there is currently an absence of reliable biomarkers of PNS injury.^8^ Thus, we have focused on the clinical and research advantages of using BBs in conjunction with other diagnostic tools to provide a better understanding of CNS brain injury associated with SARS-CoV-2 and, potentially, with its ongoing mutations.

Viral mutations present an especially worrisome challenge to responses to infection by the SARS-CoV-2 virus. There are 219 viruses currently known to be capable of infecting humans, and microbes continuously mutate to enhance their capabilities for human infection. The mutation spike D614G in SARS-CoV-2 has been described as “of urgent concern” given that it represents the emergence of a dominant and more transmissible form of the virus. Some investigators have reported that the spike protein D614G and RdRp P323L mutations in SARS-CoV-2 are associated with severity of COVID-19.^9^ Other data show that, over the course of only a month, the variant carrying the more infectious D614G spike mutation became the globally dominant form of SARS-CoV-2. D614G is associated with potentially higher viral loads in COVID-19 patients, but not injury severity.^9^ Similar observations were made in Houston, Texas. Between the first wave of infection in April 2020 and the second wave in June 2020, the more infectious, but not more virulent, D614G mutation increased from 71% to 99.9% of infections, completing its domination of this local outbreak.^10^

Secondary infections have been reported internationally. A case report documented reinfection by a variant of the SARS-CoV-2 that had significantly different genomic sequences from the variant causing the first infection.^11,12^ Significantly, the SARS-CoV-2 reinfection resulted in a worse disease progression than the first infection. In addition, a prevalent Eurasian avian-like H1N1 swine influenza virus possesses 2009 pandemic genes facilitating human infection.^13^ The mutability of SARS-CoV-2 has prompted investigators to speculate that the virus will be a permanent addition to viruses that can infect humans (e.g., https://www.bbc.com/news/uk-53875189), as has been the case for four other, less virulent coronaviruses associated with 15% of common colds.

The mutability of the spike protein and its potential for rapid spread should alert scientists and public health experts to the possible appearance of spike mutations favoring CNS infection given that sequestration in the CNS confers survival advantages by protecting viruses from systemic immune responses. An analysis pipeline has been developed to facilitate real-time mutation tracking in SARS-CoV-2, focusing initially on the spike protein because it mediates infection of human cells and is the target of most vaccine strategies and antibody-based therapeutics.^14^ To date, the pipeline has identified 14 mutations in spike that are accumulating. Such mutations, considered in a broader phylogenetic context, provide an early warning system to reveal mutations that may confer selective advantages in transmission or resistance to interventions. Each mutation is evaluated for evidence of positive selection, and the implications of the mutation are explored through structural modeling. We encourage, where possible, the screening and evaluation process to include assessments of the potential of spike protein mutations to favor CNS infection. Fortunately, the technology we propose here has the potential for detecting CNS damage resulting from any viral infection.

## Clinical Presentations

Although the novel SARS-CoV-2 virus is primarily associated with respiratory problems, investigators are becoming increasingly aware of extrapulmonary complications of COVID-19, including CNS pathologies^15^ (for recent reviews, see previous works^16–19^). Table 1A summarizes international reports of neurological symptoms, and Table 1B summarizes pathological observations in COVID-19 patients, as of September 2020.

**Table 1.** CNS Injury in COVID-19 Patients A. CNS Injury in COVID-19 Patients: Neurological Manifestations

Category	Type of study (location)	Patient population and characteristic	Description of neurological manifestation		No. of patients (percentage)
Neurological symptoms	Prospective, multi-center observational study (Manhattan, Brooklyn, and Mineola, New York, USA)^20^	4491 hospitalized patients with COVID-19	Neurological disorder (toxic/metabolic encephalopathy, stroke, [ischemic, ICH/IVH, or spontaneous SAH], hypoxic/ischemic brain injury, seizure, neuropathy (including Guillain-Barre syndrome), myopathy, movement disorder, encephalitis, meningitis, myelitis, and myelopathy)	In a median of 2 days from COVID-19 symptom onset	606 (13.5%)
				Pre-admit or at time of admission^b^	419 (9.3%)
				Post-admission^b^	180 (4%)
	Observational multi-center study (Hubei province, Sichuan province, and Chongqing municipality, China)^21^ (additional study results were presented elsewhere^22^)	917 patients with COVID-19	New-onset neurological events based on manifestations, clinical examination, and investigations		39 (4.3%)
			Critical neurological events including disorders of consciousness, stroke, CNS infection, seizures, and status epilepticus		32 (3.5%) including 30 of 319 (9.4%) severe cases
			Non-critical neurological event		7 (<1%)
	Retrospective, multi-center observational study (Brescia, Novara, and Sassari, Italy)^23^	725 consecutive hospitalized patients with COVID-19	Acute neurological symptoms requiring neuroimaging; no additional details		108 (15%)
	Retrospective, multi-center observational study (Chicago, Illinois, USA)^24^	509 consecutive hospitalized patients with COVID-19	Neurological manifestations	Any time during the disease course	419 (82.3%)
				At COVID-19 onset	215 (42.2%)
				At hospitalization	319 (62.7%)
			No. of neurological manifestations (any time during the disease course)	0	77 (15.1%)
				1	146 (28.7%)
				2	133 (26.1%)
				3	101 (19.8%)
				≥4	52 (10.2%)
	Retrospective, single-center observational case study (New York, New York, USA)^25^	242 patients with COVID-19 who underwent CT or MRI for clinical indications	“Focal neurological deficits”; no additional information provided		30 (12.4%)
	Prospective, observational study (Ankara, Turkey)^26^	239 patients with COVID-19 (neuroimaging techniques were either not performed or were limited in the epidemic period of COVID-19)	Neurological findings		83 (34.7%)
	Retrospective, multi-center observational study (Istanbul, Turkey)^27^	235 ICU patients (subpopulation of 749 inpatients with COVID-19)	“Neurological symptoms”; no additional information provided		50 (31%)
	Retrospective, observational case series (Wuhan, China)^4^	214 consecutive patients with ARDS attributable to COVID-19	Total nervous system symptoms		78 (36.4%)
			CNS manifestations (i.e., dizziness, headache, impaired consciousness, acute cerebrovascular disease, ataxia, or seizure)		53 (24.8%)
	Prospective study (Fuyang, China)^28^	60 recovered COVID-19 patients 39 age- and sex-matched non-COVID-19 controls	Neurological symptoms	Acute stage	41 (68.3%)
			Neurological symptoms	Follow-up	33 (55.0%)
	Observational (Strasbourg, France)^29^	58 of 64 consecutive patients with ARDS attributable to COVID-19	Neurological findings (e.g., positive findings on CAM-ICU, agitation, corticospinal tract signs, or dysexecutive syndrome)	On admission (before treatment)	8 (14%)
				After sedation and a neuromuscular blocker were withheld	39 (67%)
	Case-series study (London, UK)^6^	27 children with COVID-19 and pediatric multi-system inflammatory syndrome	New-onset neurological symptoms		4 (14.8%)
	Case report (Philadelphia, Pennsylvania, USA)^30^	2 patients with COVID-19	Patients had concurrent neurological symptoms who repeatedly tested negative for SARS-CoV-2 RNA in their CSF.		2
Seizure/status epilepticus	Prospective, multi-center observational study (Manhattan, Brooklyn, and Mineola, New York, USA)^20^	4491 hospitalized patients with COVID-19	Seizure (clinical or electrographic)	In a median of 2 days from COVID-19 symptom onset	74 (1.6%)
				Pre-admit or at time of admission^b^	38 (0.8%)
				Post-admission^b^	29 (0.6%)
	Retrospective, single-center observational case study (Castilla-La Mancha, Spain)^7^	841 patients hospitalized with COVID-19	Seizures		6 (0.7%)
	Retrospective, multi-center observational study (Chicago, Illinois, USA)^24^	509 consecutive hospitalized patients with COVID-19	Seizures	Any time during the disease course	4 (0.8%)
				At COVID-19 onset	2 (0.4%)
	Retrospective, multi-center study (Hubei province, Sichuan province, and Chongqing municipality, China)^22^ (additional study results were presented elsewhere^21^)	304 consecutive hospitalized patients from 42 hospitals	Seizure-like events detected in 2 patients, possibly caused by acute stress reaction and hypocalcemia, respectively		2 (<1%)
	Retrospective study (New Orleans, Louisiana, USA)^31^	250 COVID-19 patients; 80% were African American and had hypertension (79%)	Seizure	At presentation	1 (<1%)
			Seizure	During hospitalization	10 (4%)
			Status epilepticus	During hospitalization	1 (<1%)
	Retrospective, observational case series (Wuhan, China)^4^	214 consecutive patients with ARDS attributable to COVID-19	Seizures		1 (0.5%)
	Observational (Kirkland, Washington, USA)^32^	21 patients with COVID-19	Seizures		1 (4.8%)
	Case series (Granada, Italy)^33^	6 patients with COVID-19 and ischemic stroke confirmed with CT	Seizures		1 (17%)
	Case series (Stanford, California, USA)^34^	5 critically ill adult patients with COVID-19 who underwent EEG monitoring	Seizure-like movements		3
			Status epilepticus		2
	Case report (Lausanne, Switzerland)^35^	2 patients with COVID-19	Status epilepticus		1
	Retrospective, single-center case series (Birmingham, Alabama, USA)^36^	2 patients with COVID-19	*De novo* status epilepticus		2
	Case series (Cleveland, Ohio, USA)^37^	2 patients with COVID-19	Acute symptomatic seizures		1
			Acute symptomatic seizures/status epilepticus		1
	Case report (Geneva, Switzerland)^38^	1 patient with COVID-19-related ARDS	Non-convulsive status epilepticus detected by EEG (CSF SARS-CoV-2 test was negative)		1
	Case report (Los Angeles, California, USA)^39^	1 patient with COVID-19 without respiratory failure	Seizures		1
	Case report (Brescia, Italy)^40^	1 patient with COVID-19 admitted for interstitial pneumonia and seizures	Seizures (CSF RT-PCR for neurotropic viruses, including SARS-CoV-2, was negative)		1
	Case report (Sari, Iran)^41^	1 patient with COVID-19	Seizures (CSF sample was unremarkable for COVID-19 infection, brain MRI was normal)		1
	Case report (Brooklyn, New York, USA)^42^	1 patient with COVID-19	Status epilepticus		1
	Case report (Brooklyn, New York, USA)^43^	1 patient with COVID-19	Seizures		1
	Case report (Nantes, France)^44^	1 patient with COVID-19	Non-lesional status epilepticus		1
	Case report (Modena, Italy)^45^	1 patient with COVID-19	Motor seizures, brain CT was normal		1
	Case report (Tehran, Iran)^46^	1 patient with COVID-19	Generalized tonic-clonic seizures		1
	Case report (Yamanashi, Japan)^47^	1 patient with COVID-19	Seizures		1
	Case report (Samsun, Turkey)^48^	1 patient with COVID-19	Seizures		1
	Case report (Tehran, Iran)^49^	1 patient with COVID-19	Lethal status epilepticus		1
	Case report (London, United Kingdom)^50^	1 patient with COVID-19	Seizures		1
Impaired consciousness	Observational, multi-center study (Hubei province, Sichuan province, and Chongqing municipality, China)^21^ (additional study results were presented elsewhere^22^)	917 patients with COVID-19	Conscious disturbance (1 patient had TBI)		25 (2.7%)
			Impaired consciousness		25 (2.7%)
	Retrospective, single-center observational case study (Castilla-La Mancha, Spain)^7^	841 patients hospitalized with COVID-19	Disorders of consciousness		165 (19.6%)
			Syncope		5 (0.6%)
	Retrospective, multi-center observational study (Chicago, Illinois, USA)^24^	509 consecutive hospitalized patients with COVID-19	Syncope	Any time during the disease course	15 (4%)
				At COVID-19 onset	6 (1.2%)
	Retrospective study (New Orleans, Louisiana, USA)^31^	250 COVID-19 patients; 80% were African American and had hypertension (79%)	Syncope	At presentation	6 (2%)
	Prospective, observational study (Ankara, Turkey)^26^	239 patients with COVID-19 (neuroimaging techniques were either not performed or were limited in the epidemic period of COVID-19)	Impaired consciousness-confusion		23 (9.6%)
	Retrospective, observational case series (Wuhan, China)^4^	214 consecutive patients with ARDS attributable to COVID-19	Impaired consciousness		16 (7.5%)
	Retrospective, multi-center observational cohort study (France)^51^	37 patients from a cohort of 190 consecutive patients with severe COVID-19, neurological manifestation, and abnormal MRI findings (excluding ischemic stroke)	Alteration of consciousness		27 (37.73%)
			Confusion		12 (37.32%)
	Case report (Philadelphia, Pennsylvania, USA)^30^	2 patients with COVID-19	Loss of consciousness		1
	Case report (Yamanashi, Japan)^47^	1 patient with COVID-19	Consciousness disturbance		1
	Case report (Dubai, United Arab Emirates)^52^	1 patient with COVID-19	Drowsiness and mild confusion		1
	Case report (London, UK)^50^	1 patient with COVID-19	Reduced level of consciousness		1
**Headache**	Retrospective, single-center observational case study (Castilla-La Mancha, Spain)^7^	841 patients hospitalized with COVID-19	Headache		119 (14.1%)
	Observational, multi-center study (Hubei province, Sichuan province, and Chongqing municipality, China)^21^ (additional study results were presented elsewhere^22^)	917 patients with COVID-19	Unexplained headache		2 (<1%)
	Retrospective, multi-center observational study (Chicago, Illinois, USA)^24^	509 consecutive hospitalized patients with COVID-19	Headache	Any time during the disease course	192 (37.7%)
				At COVID-19 onset	84 (16.5%)
	Retrospective study (New Orleans, Louisiana, USA)^31^	250 COVID-19 patients; 80% were African American and had hypertension (79%)	Headache	At presentation	6 (2%)
			Headache	During hospitalization	19 (8%)
	Prospective, observational study (Ankara, Turkey)^26^	239 patients with COVID-19 (neuroimaging techniques were either not performed or were limited in the epidemic period of COVID-19)	Headache		64 (26.7%)
	Retrospective, observational case series (Wuhan, China)^4^	214 consecutive patients with ARDS attributable to COVID-19	Headache		28 (13.1%)
	Observational/retrospective questionary (Rome, Italy)^53^	143 recovered COVID-19 patients	Headache	Acute phase of COVID-19	∼49%
				Follow-up	∼9%
	Case-series study (London, UK)^6^	27 children with COVID-19 pediatric multi-system inflammatory syndrome	Headache		3 (11%)
	Case report (Philadelphia, Pennsylvania, USA)^30^	2 patients with COVID-19 and stroke	Acute-onset severe headache		1
	Case report (Istanbul, Turkey)^54^	1 patient with COVID-19 and encephalomyelitis confirmed with MRI	Persistent headache		1
	Case report (Brescia, Italy)^40^	1 patient with COVID-19 admitted for interstitial pneumonia and seizures	Headache		1
	Case report (Samsun, Turkey)^48^	1 patient with COVID-19	Headache		1
	Case report (London, UK)^50^	1 patient with COVID-19	Headache		1
	Case report (Recife, Brazil)^55^	1 COVID-19 patient	Severe and persistent headache		1
Altered mental status	Retrospective study (New Orleans, Louisiana, USA)^31^	250 COVID-19 patients; 80% were African American and had hypertension (79%)	Altered mental status	At presentation	19 (8%)
				During hospitalization	73 (29%)
	UK-wide surveillance study (United Kingdom)^5^	125 (82%) of 153 patients with broad clinical syndromes associated with COVID-19	Altered mental status	Total	39 (31%)
				Neuropsychiatric disorder	23 (59%)^a^, including 21 (92%) new cases
	Retrospective, multi-center observational study (Brescia, Novara, and Sassari, Italy)^23^	108 (15%) patients (subpopulation of 725 consecutive hospitalized patients with COVID-19) who had acute neurological symptoms requiring neuroimaging (CT and/or MRI)	Altered mental status		64 (59%)
Altered sense of smell (anosmia) or taste (ageusia/dysgeusia)	Retrospective, single-center observational case study (Castilla-La Mancha, Spain)^7^	841 patients hospitalized with COVID-19	Anosmia		41 (4.9%)
			Dysgeusia		52 (6.2%)
	Retrospective, multi-center observational study (Chicago, Illinois, USA)^24^	509 consecutive hospitalized patients with COVID-19	Anosmia	Any time during the disease course	58 (11.4%)
				At COVID-19 onset	18 (3.5%)
			Dysgeusia	Any time during the disease course	81 (15.9%)
				At COVID-19 onset	24 (4.7%)
	Retrospective study (New Orleans, Louisiana, USA)^31^	250 COVID-19 patients; 80% were African American and had hypertension (79%)	Ageusia	At presentation	1 (<1%)
			Ageusia/anosmia	During hospitalization	3 (1%)
	Prospective, observational study (Ankara, Turkey)^26^	239 patients with COVID-19 (neuroimaging techniques were either not performed or were limited in the epidemic period of COVID-19)	Smell impairment		18 (7.5%)
			Taste impairment		16 (6.7%)
	Retrospective, observational case series (Wuhan, China)^4^	214 consecutive patients with ARDS attributable to COVID-19	Taste impairment		12 (5.6%)
			Smell impairment		11 (5.1%)
	Cross-sectional, survey-based study (Treviso, Italy)^56^	202 mildly symptomatic patients with COVID-19	Altered sense of smell or taste		113 (55.6%)
	Observational/retrospective questionnaire (Rome, Italy)^53^	143 recovered COVID-19 patients	Anosmia	Acute phase of COVID-19	∼43%
				Follow-up	∼15%
	Case report (Istanbul, Turkey)^54^	1 patient with COVID-19 and encephalomyelitis confirmed with MRI	Anosmia		1
	Case report (Brescia, Italy)^40^	1 patient with COVID-19 admitted for interstitial pneumonia and seizures	Anosmia and ageusia (CSF RT-PCR for neurotropic viruses, including SARS-CoV-2, was negative)		1
	Case report (Recife, Brazil)^55^	1 COVID-19 patient	Anosmia		1
			Ageusia		1
Neuropsychiatric disturbances	Retrospective, single-center observational case study (Castilla-La Mancha, Spain)^7^	841 patients hospitalized with COVID-19 (electronic medical records, laboratory parameters, radiological examinations [head CT and/or brain MRI], and neurophysiological tests, including EEG and EMG, if indicated)	Neuropsychiatric disorder (insomnia, depression, anxiety, or psychosis)		167 (19.9%)
			Insomnia		109 (13%)
			Anxiety		68 (8.1%)
			Depression		44 (5.2%)
			Psychosis		11 (1.3%)
	Prospective cohort study (Milan, Italy)^57^	402 adults COVID-19 survivors with psychiatric symptoms enrolled during an ongoing prospective cohort study at IRCCS San Raffaele Hospital. The results are based on a clinical interview and a battery of self-report questionnaires.	At least one psychiatric symptom		56%
			Anxiety		42%
			Insomnia		40%
			Depression		31%
			PTSD		28%
			Obsessive-compulsive symptomatology		20%

**Table:** B. CNS Injury in COVID-19 Patients: Cerebrovascular and Other Neuropathological Observations

Category	Type of study (location)	Patient population and characteristic	Description of neurological manifestation		No. of patients (percentage)
Cerebrovascular injury	Prospective, multi-center observational study (Manhattan, Brooklyn, and Mineola, New York, USA)^20^	4491 hospitalized patients with COVID-19	Stroke (any type)	In a median of 2 days from COVID-19 symptom onset	84 (1.9%)
				Pre-admit or at time of admission^b^	33 (0.7%)
				Post-admission^b^	25 (0.6%)
			Ischemic/TIA	In a median of 2 days from COVID-19 symptom onset	61 (1.4%)
				Pre-admit or at time of admission^b^	37 (0.8%)
				Post-admission^b^	43 (1%)
			ICH/IVH	In a median of 2 days from COVID-19 symptom onset	20 (0.4%)
				Pre-admit or at time of admission^b^	3 (0.1%)
				Post-admission^b^	17 (0.4%)
			Spontaneous SAH	In a median of 2 days from COVID-19 symptom onset	3 (0.1%)
				Pre-admit or at time of admission^b^	0 (0%)
				Post-admission^b^	2 (<0.1%)
	Multi-center, retrospective cohort study (New York, New York, USA)^58^	2132 patients with emergency department visits or hospitalizations with COVID-19	Ischemic stroke (imaging for diagnosis, CT and/or MRI)		31 (1.5%)
	Retrospective study (Albacete, Spain)^59^	1683 consecutive admissions with COVID-19	Cerebrovascular disease confirmed with CT and MRI in selected cases, total		23 (1.4%)
			Cerebral ischemia		17 (73.9%)^a^
			Cerebral macrohemorrhages		5 (21.7%)^a^
	Single-center, retrospective, observational study (Madrid, Spain)^60^	1200 patients with COVID-19	Ischemic stroke confirmed with CT		8 (0.7%)
	Observational, multi-center study (Hubei province, Sichuan province, and Chongqing municipality, China)^21^ (additional study results were presented elsewhere^22^)	917 patients with COVID-19	Stroke		10 (1.1%)
	Retrospective, single-center observational case study (Castilla-La Mancha, Spain)^7^	841 patients hospitalized with COVID-19 (electronic medical records, laboratory parameters, radiological examinations [head CT and/or brain MRI], and neurophysiological tests, including EEG and EMG, if indicated)	Ischemic stroke		11 (1.3%)
			Intracranial hemorrhage		3 (0.4%)
	Retrospective, observational cohort study (New York, New York, USA)^61^	755 patients COVID-19 who underwent CT and/or MRI neuroimaging	Intracranial hemorrhage		37 (4.9%)
	Retrospective, observational cohort study (New York, New York, USA)^61^	755 patients COVID-19 who underwent CT and/or MRI neuroimaging	Intracranial hemorrhage without evidence of secondary to trauma or brain metastases		33 (4.4%)
	Retrospective, multi-center observational study (Chicago, Illinois, USA)^24^	509 consecutive hospitalized patients with COVID-19	Ischemic stroke	Any time during the disease course	7 (1.4%)
				At COVID-19 onset	0 (0%)
			Hemorrhagic stroke	Any time during the disease course	1 (0.3%)
				At COVID-19 onset	0 (0%)
	Observational, single-center study (Milan, Italy)^62^	388 ICU patients with COVID-19	Ischemic stroke as reported by the treating physicians in the medical charts		9 (2.5%)
	Retrospective study (New Orleans, Louisiana, USA)^31^	250 COVID-19 patients; 80% were African American and had hypertension (79%)	Cerebrovascular accident during hospitalization		1 (<1%)
	Retrospective, single-center observational case study (New York, New York, USA)^25^	242 patients with COVID-19 who underwent CT or MRI for clinical indications	Non-specific white matter microangiopathy		123 (50.8%)
			Chronic infarct		47 (19.4%)
			Subacute ischemic infarct		13 (5.4%)
			Acute hemorrhage		11 (4.5%)
	Prospective, observational study (Ankara, Turkey)^26^	239 patients with COVID-19 (neuroimaging techniques were either not performed or were limited in the epidemic period of COVID-19)	Cerebrovascular disorders	Total	9 (3.8%)
				Ischemic	7 (2.9%)
				Hemorrhagic	2 (0.8%)
	Single-center, retrospective, observational study (Wuhan, China).^63^ The results, in part, were also presented elsewhere.^4^	219 patients with COVID-19	Acute cerebrovascular disease diagnosed by clinical symptoms and head CT	Ischemic stroke	10 (4.6%)
				ICH	1 (0.5%)
	Retrospective, observational case series (Wuhan, China)^4^	214 consecutive patients with ARDS attributable to COVID-19	Acute cerebrovascular disease includes ischemic stroke and cerebral hemorrhage diagnosed by clinical symptoms and head CT.		6 (2.8%)
	Multi-center cohort study (The Netherlands)^64,65^	184 ICU patients with proven COVID-19 pneumonia	Arterial thrombotic events/ischemic stroke diagnosed with CT	Follow-up observation duration 7 days (median)	3 (1.6%)
				Follow-up observation duration 14 days (median)	5 (2.7%)
	UK-wide surveillance study (United Kingdom)^5^	125 (82%) of 153 patients with broad clinical syndromes associated with COVID-19	Cerebrovascular event	Total	77 (62%)
				Ischemic stroke	57 (74%)^a^
				ICH	9 (12%)^a^
				CNS vasculitis	1 (1%)^a^
				Other cerebrovascular events	10 (13%)^a^
	Retrospective, multi-center observational study (Brescia, Novara, and Sassari, Italy)^23^	108 (15%) patients (subpopulation of 725 consecutive hospitalized patients with COVID-19) who had acute neurological symptoms requiring neuroimaging (CT and/or MRI)	Acute ischemic infarcts		34 (31%)
			Intracranial hemorrhages		6 (6 %)
			Cerebral venous thrombosis		2 (2%)
	Observational single-center study (Cambridge, UK)^66^	63 patients with COVID-19	Composite end point included arterial thrombosis (myocardial infarction, stroke, or peripheral artery embolism) with no additional details.		4% (cumulative incidence estimate)
	Retrospective, observational study (London, UK)^67^	43 patients with COVID-19 referred to the neurology/encephalitis and neurovascular multi-disciplinary teams meetings	Ischemic strokes associated with a prothrombotic state (imaging for diagnosis, MRI and/or CT)		8 (18.6%)
	Retrospective, observational single-center study (Paris, France)^68^	37 consecutive patients with acute ischemic stroke attributable to large-vessel occlusion	Large-vessel occlusion with positive COVID-19 test diagnosed by MRI or CT imaging		10 (27%)
	Observational, multi-center study (Hubei province, Sichuan province, and Chongqing municipality, China)^21^ (additional study results were presented elsewhere^22^)	28 patients with COVID-19 (subpopulation of 917) who underwent brain CT	Stroke	New onset	7 (25%)
			Stroke	Previous history, total	4 (14%)
			Stroke	Previous history with new lesions	1 (4%)
	Retrospective, multi-center observational cohort study (France)^51^	37 patients from a cohort of 190 consecutive patients with severe COVID-19, neurological manifestation, and abnormal MRI findings (excluding ischemic stroke)	Intracerebral hemorrhagic lesions Including:		20 (54%)
	Single-center, retrospective, observational study (Abu Dhabi, United Arab Emirates)^69^	20 patients with COVID-19 and acute ischemic stroke	Large-vessel occlusion		15 (75%)
	Observational (Strasbourg, France)^29^	13 patients (subpopulation of 58) who underwent MRI even though they did not have focal signs that suggested stroke	Ischemic stroke		3 (23%)
	Single-center observational case study (Brescia, Italy)^70^	11 critically ill patients with COVID-19 with persistently depressed mental status who underwent MRI	Confluent T_2_ hyperintensity and mild restricted diffusion in bilateral supratentorial deep and subcortical white matter		10 (91%)
			Multiple punctate microhemorrhages in juxtacortical and callosal white matter		7 (64%)
	Retrospective, observational, single-center study (New York, USA)^71^	10 consecutive patients who underwent endovascular stroke thrombectomy	Emergent large-vessel occlusion with positive COVID-19 test		5 (50%)
	Case series (Granada, Italy)^33^	6 patients with COVID-19	Ischemic stroke confirmed with CT		6
			Ischemic stroke confirmed with CT		6
	Case series (New York, New York, USA)^72^	5 patients with COVID-19	Large-vessel stroke detected by CT and/or MRI NIHSS on admission: 13–23 (range) NIHSS at 24 h: 4–19 (range)		5
	Case series (New York, New York, USA)^73^	5 consecutive patients with COVID-19 who underwent endovascular thrombectomy to treat large-vessel occlusions	Acute stroke attributable to large-vessel occlusions presented with coagulation abnormalities		5
	Case series (Granada, Spain)^74^	4 patients with COVID-19	Ischemic stroke confirmed with CT		4
	Retrospective case series (United States)^75^	4 patients with COVID-19	Ischemic stroke confirmed with CT		4
	Case report (Wuhan, China)^76^	3 patients with COVID-19 and clinically significant coagulopathy, antiphospholipid antibodies, and multiple infarcts	Multiple cerebral infarctions confirmed by CT imaging in all patients		3
	Case series (Brooklyn, New York, USA)^77^	3 patients with COVID-19	Intraparenchymal hemorrhage confirmed with CT		1
	Case series (New York, New York, USA)^78^	3 patients with COVID-19	Ischemic stroke confirmed with CT		1
	Case series (New York, New York, USA)^78^	3 patients with COVID-19	Acute/subacute infarct within thalamus		1
	Case report (Bronx, New York, USA)^79^	3 critically ill patients with COVID-19 on therapeutic anticoagulation	Intracranial hemorrhage confirmed with CT		3
	Case series (Newark, New Jersey, USA)^80^	2 pediatric patients with orbital cellulitis, sinusitis, and COVID-19	Intracranial hemorrhagic abnormalities confirmed with CT		2
	Case report (Philadelphia, Pennsylvania, USA)^30^	2 patients with COVID-19	Hunt and Hess grade 3 aneurysmal SAH (imaging for diagnosis: CT)		1
	Case report (United States)^81^	2 patients with COVID-19	Hemorrhagic posterior reversible encephalopathy syndrome diagnosis detected by CT and MRI		2
	Case report (Philadelphia, Pennsylvania, USA)^30^	2 patients with COVID-19	Ischemic stroke (imaging for diagnosis, CT)		1
	Case report (Trévenans, France)^82^	2 patients with COVID-19	Acute ischemic stroke in multiple vascular areas diagnosed with MRI; presumed thrombotic stroke that occurred during ongoing anticoagulation treatment		2
	Case report (New York, New York, USA)^83^	2 patients with COVID-19	Ischemic stroke attributable to acute thrombosis in the ipsilateral common carotid artery bifurcation confirmed with MRI and CT		2
	Case series (Philadelphia, Pennsylvania, USA)^84^	2 critically ill patients with COVID-19	Intracranial hemorrhage confirmed with CT		2
	Case report (New York, New York, USA)^85^	2 critically ill patients with COVID-19	Intracranial hemorrhage confirmed with CT		2
	Case report (Geneva, Switzerland)^38^	1 patient with COVID-19-related ARDS	Cerebral microbleeds in white matter detected by MRI (CSF SARS-CoV-2 test was negative)		1
	Case report (Bilbao, Spain)^86^	1 patient with COVID-19	Ischemic stroke confirmed with CT		1
	Case report (Strasbourg, France)^87^	1 patient with COVID-19	SAH confirmed with CT		1
	Case report (Baltimore, Maryland, USA)^88^	1 patient with COVID-19	Ischemic stroke confirmed with CT		1
	Case report (Flint, Michigan, USA)^89^	1 patient with COVID-19	Ischemic stroke confirmed with CT		1
	Case report (Sari, Iran)^90^	1 patient with COVID-19	ICH diagnosis detected by CT imaging		1
	Case report (Wuhan, China)^91^	1 patient with COVID-19	Cerebral hemorrhage confirmed with CT		1
	Case report (Isfahan, Iran)^92^	1 patient with COVID-19	Cerebral venous thrombosis/hemorrhagic infarct confirmed with CT		1
	Case report (Singapore)^93^	1 patient with COVID-19	Intracranial hemorrhage confirmed with CT		1
	Case report (Manila, Philippines)^94^	1 patient with COVID-19	Ischemic stroke confirmed with CT		1
	Case report (Fortaleza, Brazil)^95^	1 patient with COVID-19	Ischemic stroke confirmed with CT		1
	Case report (Düsseldorf, Germany)^96^	1 patient with COVID-19	SAH attributed to a ruptured pericallosal artery aneurysm confirmed with CT		1
	Case report (Maastricht, The Netherlands)^97^	1 patient with COVID-19	Ischemic stroke confirmed with CT		1
	Case report (Brooklyn, New York, USA)^43^	1 patient with COVID-19	Head CT showed chronic microvascular ischemic changes, but did not show any acute changes, infarct, or hemorrhage.		1
	Case report (Dubai, United Arab Emirates)^52^	1 patient with COVID-19	Meningoencephalitis complicated with intracranial hemorrhage confirmed with CT		1
	Case report (Southampton, UK)^98^	1 patient with COVID-19	Cerebral CNS inflammatory vasculopathy with antimyelin oligodendrocyte glycoprotein antibodies detected by MRI		1
	Case report (Rome, Italy)^99^	1 patient with COVID-19	Deep intracerebral venous thrombosis confirmed with CT		1
Encephalopathy	Prospective, multi-center observational study (Manhattan, Brooklyn, and Mineola, New York, USA)^20^	4491 hospitalized patients with COVID-19	Toxic/metabolic encephalopathy	In a median of 2 days from COVID-19 symptom onset	309 (6.8%)
				Pre-admit or at time of admission^b^	240 (5.4%)
				Post-admission^b^	71 (1.6%)
	Retrospective study (Albacete, Spain)^59^	1683 consecutive admissions with COVID-19	Leukoencephalopathy of posterior reversible encephalopathy type		1 (<1%)
	Retrospective, multi-center observational study (Chicago, Illinois, USA)^24^	509 consecutive hospitalized patients with COVID-19	Encephalopathy	Any time during the disease course	162 (31.8%)
				At COVID-19 onset	9 (1.8%)
	UK-wide surveillance study (United Kingdom)^5^	125 (82%) of 153 patients with broad clinical syndromes associated with COVID-19	Unspecified encephalopathy		9 (7.2%)
	Retrospective, observational study (London, UK)^67^	43 patients with COVID-19 referred to the neurology/encephalitis and neurovascular multi-disciplinary teams meetings	Encephalopathies with delirium/psychosis and no distinct MRI or CSF abnormalities		10
	Case series (Stanford, California, USA)^34^	5 critically ill adult patients with COVID-19 who underwent EEG monitoring	Encephalopathy		5
	Case report (Atlanta, Georgia, USA)^100^	3 patients with COVID-19	Encephalopathy diagnosis detected by neurological examination and MRI		2
	Case series (Cambridge, Massachusetts, USA)^101^	2 patients with COVID-19	Posterior reversible encephalopathy syndrome diagnosis detected by CT and MRI		2
	Case report (Boca Raton, Florida, USA)^102^	1 patient with COVID-19	Encephalopathy CT scan of the head was consistent with the previous history of embolic stroke, but showed no acute abnormalities.		1
	Case report (Detroit, Michigan, USA)^103^	1 patient with COVID-19	Acute hemorrhagic necrotizing encephalopathy diagnosis detected by CT and MRI imaging		1
	Case report (Boca Raton, Florida, USA)^104^	1 patient with COVID-19	Encephalopathy confirmed by MRI		1
	Case report (Varese, Italy)^105^	1 patient with COVID-19	Posterior reversible encephalopathy syndrome diagnosis detected by CT and MRI		1
	Case report (Uppsala, Sweden)^106^	1 patient with COVID-19	Acute necrotizing encephalopathy with diagnosis detected by MRI		1
	Case report (London, UK)^50^	1 patient with COVID-19	Acute hemorrhagic necrotizing encephalopathy with diagnosis detected by CT and MRI		1
Encephalitis	Prospective, multi-center observational study (Manhattan, Brooklyn, and Mineola, New York, USA)^20^	4491 hospitalized patients with COVID-19	Encephalitis referable to SARS-CoV-2 infection in a median of 2 days from COVID-19 symptom onset		0 (0%)
	Retrospective, single-center observational case study (Castilla-La Mancha, Spain)^7^	841 patients hospitalized with COVID-19	Encephalitis		1 (0.1%)
	Retrospective, multi-center observational study (Chicago, Illinois, USA)^24^	509 consecutive hospitalized patients with COVID-19	Encephalitis	Any time during the disease course	1 (0.2%)
				At COVID-19 onset	0 (0%)
	Retrospective study (New Orleans, Louisiana, USA)^31^	250 COVID-19 patients; 80% were African American and had hypertension (79%)	Encephalitis during hospitalization		3 (1%)
	UK-wide surveillance study (United Kingdom)^5^	125 (82%) of 153 patients with broad clinical syndromes associated with COVID-19	Encephalitis		7 (5.6%)
	Retrospective, observational study (London, UK)^67^	43 patients with COVID-19 referred to the neurology/encephalitis and neurovascular multidisciplinary teams meetings.	Encephalitis (imaging used for diagnosis, MRI and/or CT)		2
	Case report (Barcelona, Spain)^107^	2 patients with COVID-19	Encephalitis diagnosed by clinical examination and CSF analyses (brain CT and MRI scans were normal or unremarkable)		2
	Case report (Qingdao, China)^108^	1 patient with COVID-19	Viral encephalitis diagnosis based on SARS-CoV-2–positive test in CSF (no additional details)		1
	Case report (Wuhan, China)^109^	1 patient with COVID-19	Encephalitis associated with SARS-CoV-2 infection was diagnosed by neurological evaluation. The patient did not have evidence of bacterial or tuberculous infection of the CNS, and his CSF specimen was tested negative for SARS-CoV-2. Head CT imaging was normal.		1
	Case report (Brooklyn, New York, USA)^42^	1 patient with COVID-19	Encephalitis (head CT was negative)		1
	Case report (Telford, UK)^110^	1 patient with COVID-19	Rhombencephalitis/brainstem encephalitis (imaging for diagnosis, MRI)		1
	Autopsy case report (Varna, Bulgaria)^111^	1 patient with COVID-19	Acute necrotizing encephalitis		1
	Case report (Brescia, Italy)^112^	1 patient with COVID-19	Encephalitis diagnosed by clinical examination and CSF analyses (CT and MRI were negative)		1
	Case report (Tehran, Iran)^46^	1 patient with COVID-19	Encephalitis (imaging for diagnosis, MRI)		1
	Case report (Samsun, Turkey)^48^	1 patient with COVID-19	Encephalitis mimicking a glial tumor (imaging for diagnosis, MRI)		1
	Case report (Los Angeles, California, USA)^113^	1 patient with COVID-19	Encephalitis		1
Encephalomyelitis	Case report (Tehran, Iran)^114^	1 patient with COVID-19	Encephalomyelitis confirmed with MRI		1
	Case report (Istanbul, Turkey)^54^	1 patient with COVID-19	Encephalomyelitis confirmed with MRI		1
Meningoencephalitis	Prospective, multi-center observational study (Manhattan, Brooklyn, and Mineola, New York, USA)^20^	4491 hospitalized patients with COVID-19	Encephalitis/meningitis referable to SARS-CoV-2 infection in a median of 2 days from COVID-19 symptom onset		0 (0%)
	Case series (Istanbul, Turkey)^115^	29 intubated patients with COVID-19	Meningoencephalitis with pathological MRI findings		3 (10%)
			Meningoencephalitis with normal MRI		3 (10%)
	Post-mortem (Munich, Germany)^116^	6 deceased patients with COVID-19	Encephalitis/meningitis		6
	Case report (Lausanne, Switzerland)^35^	2 patients with COVID-19	Meningoencephalitis diagnosed by CSF analysis (MRI was normal)		2
	Case report (Yamanashi, Japan)^47^	1 patient with COVID-19	Meningitis/encephalitis: MRI demonstrated abnormal findings in medial temporal lobe including hippocampus, suggesting encephalitis, hippocampal sclerosis, or post-convulsive encephalitis.		1
	Case report (Los Angeles, California, USA)^39^	1 patient with COVID-19 without respiratory failure	Isolated meningoencephalitis (CT of the head without contrast was normal)		1
ADEM	Retrospective observational study (London, UK)^67^	43 patients with COVID-19 referred to the neurology/encephalitis and neurovascular multi-disciplinary teams meetings	ADEM		9
	Case report (New Britain, Connecticut, USA)^117^	1 patient with COVID-19	COVID-19-associated ADEM confirmed with MRI (SARS-CoV-2 was not detected in CSF)		1
	Case report (Tehran, Iran)^49^	1 patient with COVID-19	ADEM confirmed with MRI		1
	Case report (Rochester, Minnesota, USA)^118^	1 patient with COVID-19	ADEM-like pathology confirmed with post-mortem gross examination of the brain		1
	Case report (Genova, Italy)^119^	1 patient with COVID-19	ADEM confirmed with MRI		1
Demyelination	Prospective, multi-center observational study (Manhattan, Brooklyn, and Mineola, New York, USA)^20^	4491 hospitalized patients with COVID-19	Myelopathy/myelitis referable to SARS-CoV-2 infection in a median of 2 days from COVID-19 symptom onset		0 (0%)
	Retrospective, observational study (London, UK)^67^	43 patients with COVID-19 referred to the neurology/encephalitis and neurovascular multi-disciplinary teams meetings	Isolated myelitis (imaging for diagnosis, MRI and/or CT)		1
	Case report (Brescia, Italy)^40^	1 patient with COVID-19 admitted for interstitial pneumonia and seizures	Brain and spine demyelinating lesions detected by MRI (CSF RT-PCR for neurotropic viruses, including SARS-CoV-2, was negative)		1
Abnormal MRI findings	Prospective study (Fuyang, China)^28^	60 recovered COVID-19 patients 39 age- and sex-matched non-COVID-19 controls	MRI findings 3-month follow-up: COVID-19 patients had statistically significantly higher bilateral GMV in olfactory cortices, hippocampi, insulas, left Rolandic operculum, left Heschl's gyrus, and right cingulate gyrus vs. controls (corrected *p* value, <0.05).		
	Retrospective, multi-center observational study (Istanbul, Turkey)^27^	27 (54%) patients (subpopulation of 50 ICU patients with neurological symptoms) that underwent MRI	Abnormal MRI findings include: cortical signal abnormalities, punctate cortical blooming, pial-subarachnoid enhancement, and subcortical and deep white matter signal abnormality		12 (44%)
	Case report^120^	1 COVID-19 patient	Cytotoxic lesions of the corpus callosum detected by MRI		1
Hypoxic injury	Prospective, multi-center observational study (Manhattan, Brooklyn, and Mineola, New York, USA)^20^	4491 hospitalized patients with COVID-19	Hypoxic/ischemic brain injury in a median of 2 days from COVID-19 symptom onset		65 (1.4%)
	Post-mortem observational study (Boston, Massachusetts, USA)^121^	18 consecutive patients with COVID-19	Acute hypoxic injury in the cerebrum and cerebellum, but no thrombi or vasculitis		18 (100%)
	Case report (Atlanta, Georgia, USA)^100^	3 patients with COVID-19	Encephalitis and myelitis with superimposed hypoxic ischemic changes detected by MRI		1
Hydrocephalus	Case report (Milan, Italy)^122^	1 infant patient with COVID-19	Complex hydrocephalus with a shunt disconnection detected by CT		1

^a^ Percentage calculated from the total number of patients with a specific disorder, symptom, or neuroimaging finding.

^b^ Timing of neurological disorders missing in 7 patients.

ADEM, acute disseminated encephalomyelitis; ARDS, acute respiratory distress syndrome; CAM-ICU, confusion assessment method in the intensive care unit; CNS, central nervous system; COVID-19, coronavirus disease 2019; CSF, cerebrospinal fluid; CT, computed tomography; EEG, electroencephalography; EMG, electromyography; GMV, gray matter volume; ICH, intracerebral hemorrhage; ICU, intensive care unit; IVH, intraventricular hemorrhage; MRI, magnetic resonance imaging; NIHSS, the National Institutes of Health Stroke Scale; PTSD, post-traumatic stress disorder; RT-PCR, reverse-transcription polymerase chain reaction; SAH, subarachnoid hemorrhage; SARS-CoV-2, severe acute respiratory syndrome coronavirus 2; TIA, transient ischemic attack.

Early communications from China provided data on neurological symptoms suggesting CNS involvement in COVID-19 patients^4^ now confirmed in several other studies.^4,23,25,27,29,30,40,72^ Disturbances in taste and smell have been prominent neurological sequelae of COVID-19 infection,^56^ but neurological symptoms can also include descriptions of impaired consciousness and confusion, altered mental status, confusion, headache, and syncope as well as anxiety, depression, and post-traumatic stress disorder (PTSD).^7,57^ Neurological manifestations have been observed in 42% of COVID-19 patients at disease onset, 63% during hospitalization, and 82% at some time during the course of the disease.^24^ In contrast, a prospective study of hospitalized COVID-19 patients reported that 13.5% developed new neurological symptoms before or during hospitalization with a median onset of 2 days from COVID-19 symptoms onset.^20^ These data signal the importance of standardizing the evaluation of neurological assessments across study sites and, where possible, linking behavioral observations to organically based assessments such as BBs and imaging.

There are also multiple descriptions of CNS pathological observations that include a predominance of reports of cerebrovascular injury, such as diffuse cerebral ischemia, ischemic and hemorrhagic stroke, microhemorrhages, white matter microangiopathy, and arterial thrombosis as well as meningitis, encephalitis, acute transverse myelitis (ATM), and encephalopathy^4,20,23,24,27,29,30,39,40,67,70,72,76,90,100,102,103,109,123–126^ (see also other works^24,69,121,127–132^). Investigators have also observed associations between magnetic resonance (MRI) abnormalities and neurological deficits persisting in 55% of hospitalized patients 3 months after disease onset.^28^

Hypercoagulopathy resulting from viral effects on systemic and CNS coagulation pathways has been a growing concern, and anticoagulant administration was reported to be associated with decreased mortality in COVID-19 patients.^133^ There is also a case report of an ischemic stroke in a COVID-19 patient even though no viral particles were detected in the cerebrospinal fluid (CSF), suggesting the possibility that peripheral hypercoagulopathies could contribute to stroke in these patients.^30^

Brain hypoxia may be another prominent contributor to CNS injury, especially in patients presenting with significant pulmonary symptoms and having experienced prolonged periods of ventilator support or even extracorporeal membrane oxygenation (ECMO). “Silent hypoxemia,” oxygen levels incompatible with life without dyspnea in COVIR-19 patients, has been of concern as well, in spite of difficulties in conducting reliable assessments using pulse oximetry.^134^ Post-mortem histopathological examination of brain specimens obtained from 18 patients showed only hypoxic changes and did not detect encephalitis or other specific brain changes referable to the virus.^121^ There was no cytoplasmic viral staining, but the virus was detected at low levels in six brain sections obtained from 5 patients who were not consistently related to the interval from the onset of symptoms to death. However, the broad diagnostic categories, variability in the times of post-mortem examinations, and limited number of observations limit the generalizability of these data.

Collectively, these cases document that the CNS is among the multiple organs targeted by SARS-CoV-2. Characterization of cerebrovascular pathologies and potentially related systemic hypercoagulopathies that occur in severe COVID-19 cases are especially needed,^64^ as is a clearer understanding of the nature of persistent neurological deficits and their relationships to disease severity and epidemiological factors. In addition, longitudinal studies of affected patients should enable detection of later-emerging neurological symptoms.

## The Potential for SARS-CoV-2 to Increase Risks for Neurological Deficits and Neurodegenerative Diseases: The Need for Improved Diagnostic Rigor and Outcome Assessments

As reviewed in preceding sections and tables, a number of studies have provided evidence of CNS consequences of infection by SARS-COV-2, either by inferences from changes in neurological status or by more direct neuropathological assessments such as imaging. Studies to date have primarily been observational and used differing criteria for reporting CNS injury. For example, altered mental status is commonly used as a sign of possible CNS injury in COVID-19 patients (e.g., see earlier works^102,103,135,136^). However, functional neurological assessments can be non-specific, and neurological symptoms attributable to SARS-CoV-2 infection must be distinguished from exacerbation of pre-existing neurological and psychiatric conditions, especially in the elderly.^137,138^ Moreover, CNS pathology may not be reliably related to the severity of respiratory symptoms in COVID-19 patients.^67^ Methods to enable accurate prognoses of patients' vulnerability to neurological disturbances as well as the extent and durations of cognitive and functional deficits attributable to infection by SARS-CoV-2 are urgently needed.

The most urgent unanswered questions relate to the frequency and severity of brain injury in COVID-19 patients, identification of primary and secondary injury mechanisms contributing to this injury, risk factors for injury, and the nature and duration of neurological deficits in COVID-19 patients diagnosed with brain damage. Investigators have initiated important retrospective studies to begin to address these questions.^139^ The researchers felt, based in part on extrapolation from previous data from severe acute respiratory syndrome (SARS) and Middle East respiratory syndrome (MERS), that neurological complications in COVID-19 patients are infrequent. However, given potential that 50–80% of the world's population might be infected before herd immunity develops, they recognized the significant need for clinical, diagnostic, and epidemiological studies to characterize neurological manifestations in COVID-19 patients and the resulting disease burden.

We argue that conclusions about the frequency and severity of CNS injury and resulting neurological deficits are premature in the absence of established diagnostic and outcome measures. Our goal is to provide a framework in which such studies can be rapidly implemented to provide these much needed data. As we reference in this review, the framework incorporates technologies and clinical approaches previously successfully used in studies of other acute CNS injuries, notably TBI.

Does infection with SARS-CoV-2 increase risk for later emergence of new neurological symptoms or development of neurodegenerative diseases? Cell senescence and a stable state of proliferative arrest is an adaptive response to viral infections,^140^ and investigators have posited that SARS-CoV-2 invokes CNS cellular senescence and neurodegenerative processes.^141^ Inflammatory and neuroimmune responses to CNS infection are thought to be important mediators of Alzheimer's disease (AD).^142^ “Cytokine storms” and immunosuppression have both been reported to occur during the course of COVID-19.^143^ However, levels of inflammatory markers in COVID-19 patients may be lower than levels observed in secondary insults such as acute respiratory distress syndrome (ARDS) that could be associated with SARS-CoV-2 infection.^144^

The interleukin (IL)-6 inhibitor, tocilizumab, reduces cognitive deficits in a mouse model of AD,^145^ and IL-6 inhibitors are being studied in clinical trials for their efficacy in treating severe SARS-CoV-2 disease.^143^ Long-term follow-up of these patients will enhance understanding of the role of IL-6 and SARS-CoV-2 in any subsequent neurodegeneration. The hypothesis that initiation of AD could be attributable, in part, to systemic pathogens entering the CNS and initiating aberrant A-beta cascades^146^ or stimulating inflammation that facilitates the cascades highlights the need for such studies. There is an interesting reciprocity between COVID-19 and dementia and neurodegenerative diseases. As we document in a later section of this review, AD, Parkinson's disease (PD), and dementias are associated with more complicated clinical courses and poorer outcomes in COVID-19 patients.

## Mechanisms of Central Nervous System Injury by Coronaviruses

Viral infection of the CNS enables the pathogen to evade a response from the systemic immune systems of the host.^113^ Numerous viruses, including the coronaviruses, are known to have CNS involvement and cause brain injury after infection.^147–149^ For example, human immunodeficiency virus-1, a lentiviris,^150^ and herpes simplex virus-1, a simplexvirus,^151^ produce CNS pathologies. In fact, five neuroinvasive arboviruses have been identified as emerging potential public health threats in the United States.^152^ Zika, a flavivirus, is especially likely to cause Guillain-Barre syndrome. Chikungunya, an alphavirus, is more likely to cause inflammation and swelling in the brain (encephalitis) and spinal cord (myelitis). However, stroke, which could be caused by either virus alone, is more likely to occur in patients infected with the two viruses together.^153^

Recent studies suggest a direct CNS mechanism contributing to neurological symptoms produced by the SARS-CoV-2 virus.^154^ SARS-CoV-2, but not SARS-CoV, can infect and replicate in induced pluripotent stem cells (iPSCs)-derived human neural progenitor cells (hNPCs) and in neurospheres and brain organoids produced from these cells. Studies with the neurospheres showed that the virus is alive and able to replicate within the brain cells. The brain organoids showed morphology that was similar to developing human cerebral cortex, and this experiment showed the potential of the virus to interfere with neurogenesis. The organoids were positive for neuron-specific class III beta-tubulin (TUJ1), a marker for neuronal cells, paired box 6 (PAX6), a marker for radial glial cells, and nestin (NES), a marker of proliferation for neural progenitor cells. Brain cells have the angiotensin-converting enzyme 2 (ACE2) receptors, transmembrane serine protease 2 (TMPRSS2), cathepsin L, and furin, all of which have been identified as important for the process of infection with SARS-CoV-2 (see Zhang and colleagues 2020, supplemental material). Other investigators have confirmed that SARS-CoV-2 targets neurons of three-dimensional human brain organoids.^155^ Finally, it is important to remain vigilant to the potential for endogenous retroviruses to contribute to CNS disease.^156,157^

The ACE2 receptor plays a role in cell entry of SARs-CoV-2, similar to the other coronaviruses SARS-CoV and MERS-CoV.^158^ ACE2 receptors are present in the CNS on neurons, glia, and the cerebrovascular endothelium, the latter being one possible route of entry into the brain.^159^ Regional variability in the distribution of ACE2 receptors in the human brain has been reported. The highest ACE2 expression level was detected in the brainstem containing the medullary respiratory centers, an observation that could be relevant to the respiratory distress experienced by many COVID-19 patients.^160^ Upregulation of ACE2 in the brain has been linked to oxidative stress, apoptosis, and neuroinflammation leading to neurodegeneration in several brain disorders.^161^ Consistent with this possibility, a post-mortem case report detected SARS-CoV-2 viral particles in endothelial cells of the microvasculature of the frontal lobes.^162^

A recent case report of a patient with anosmia and confirmed SARS-CoV-2 infection described a hyperdense MRI signal on fluid-attenuated inversion recovery in the olfactory bulb and posterior gyrus rectus, a cortical region associated with olfaction, supporting the hypothesis of virus brain entry through the olfactory pathway.^163^ This potential SARS-CoV-2 entry into the CNS through the olfactory bulb is similar to the way SARS-CoV does in mice, although this remains speculative at the present time.^158,164,165^

In addition, ACE2 and TMPRSS2 have been reported to be localized to support cells, stem cells and perivascular cells rather than olfactory neurons in mouse, non-human primate, and human olfactory mucosa.^166^ Studies detecting SARS-CoV-2 in clinical specimens^167^ have shown that the highest viral copy number is found in nasal swabs (∼200-fold), compared to bronchoalveolar lavage or pharyngeal swabs. These findings, taken together with ACE2 protein cellular localization, suggest that active virus infection and replication occur in the apical layer of nasal and olfactory mucosa. The high similarities between SARS-CoV and SARS-CoV-2 has prompted some investigators to posit that the potential invasion of the CNS by SARS-CoV-2 is partially responsible for the acute respiratory failure noted in COVID-19 patients during ICU management.^168^ Others, however, suggest a more limited role of CNS involvement in the respiratory failure associated with COVID-19.^169^ Regardless, there is a need for additional studies on SARS-CoV-2 effects on the CNS, especially through the olfactory route.^165^

## Rationale for Studies of Blood Biomarkers for Diagnosis of Central Nervous System Injury: Integration with Existing Diagnostic Tools

### Use of blood biomarkers

Consistent with our understanding of evolutionary biology, proteins and cell-signaling pathways underlying CNS injury and cell death following viral infections can be interrogated by blood-based assays of relevant proteins which have already been validated extensively as biomarkers of cell death, acute brain injury and neurodegeneration. Proteins assayed by blood tests assessing mechanisms of CNS cell injury and death are highly conserved across species, including *Caenorhabditis elegans*, rodents, and humans. Table 2 summarizes human studies of brain injury BBs after systemic infections, including infections by SARS-CoV-2. A recent observational study and limited case reports detected increased CSF and/or plasma levels of glial fibrillary acidic protein (GFAP), neurofilament light polypeptide (NfL), tau, and several inflammatory markers in COVID-19 patients.^100,106,107,170,171^ Increased staining of GFAP was also detected in the post-mortem analysis of the brain of a COVID-19 patient.^118^ Studies have also reported elevations of BBs in CSF of tuberculous meningitis patients (GFAP, S100 calcium-binding protein [S100B], and neuron-specific enolase [NSE]),^172–175^ HIV patients (NfL, GFAP, and S100B),^176–180^ and cerebral malaria (S100B, NSE, tau proteins, and inflammatory protein markers).^181–186^

**Table 2.** Human Studies of Brain Injury and Inflammatory Biomarkers after COVID-19 and Other Systemic Infections

Study design	Groups (patient nos.)	Biomarkers (time points)	Major findings
Prospective cohort study^187^	Healthcare workers after a COVID-19 outbreak (*n* = 100): 1. SARS-CoV-2 positive (*n* = 28) 2. SARS-CoV-2 negative (*n* = 72)	Serum: NfL (at median of 23 days after onset of the disease and in a subset [*n* = 16] after median of 35 days)	NfL was significantly associated with COVID-19 status when controlling for age and sex. In COVID-19 patients with two NfL measurements, NfL levels were highly correlated (*r* = 0.96).
Observational cohort study^170^	1. Mild, moderate, and severe COVID-19 (*n* = 47) 2. Age-matched control from a different cohort (*n* = 33)	Plasma: GFAP, NfL (at presentation and in a subset after a mean of 11.4 days)	GFAP was increased in both severe and moderate COVID-19 cases, whereas NfL was increased only in severe cases compared to control.
Case report^112^	COVID-19 with steroid-responsive encephalitis (*n* = 1)	CSF: NfL, tau, IL-6, IL-8, TNF-α, and β2-microglobulin (3 and 10 days after admission)	Day 3: IL-6 was slightly increased, IL-8 was strongly increased, and TNF-α and β2-microglobulin were increased above the reference level. Day 10 (after steroid treatment): IL-8 and TNF-α were decreased, but remained elevated, whereas IL-6 and β2-microglobulin were stable. Tau and NfL were normal at both time points.
Case report^106^	COVID-19-related acute necrotizing encephalopathy (*n* = 1)	CSF: GFAP, NfL, tau, and IL-6 (12, 14, and 32 days after onset of COVID-19)	Day 12: GFAP, NfL, tau, and IL-6 were profoundly increased. Day 14: GFAP and IL-6 started to decrease, whereas tau and NfL increased further. Day 32: GFAP normalized, but NfL and tau remained strongly increased.
Case report^188^	Miller-Fisher syndrome after COVID-19 (*n* = 1)	Blood: NfL CSF: amyloid-β_42_, pNfH, and tau (before treatment [day 0], and 7 and 23 days)	Day 0 (before treatment): CSF pNfH was massively elevated, whereas CSF amyloid-β_42_ and tau were normal before treatment. Blood NfL was clearly increased compared to normal levels. Days 7 and 23: Blood NfL remained increased. Note: After the first CSF collection, the patient received treatment for 5 days with intravenous immunoglobulin and was free of symptoms 2 weeks after the treatment.
Case report (Atlanta, Georgia, USA)^100^	Contaminant COVID-19 infection and encephalopathy/encephalitis (*n* = 3)	CSF: inflammatory protein panel: CX3CL1, IL-10, IL12-p40, IL12-p70, IL-1α, IL-1β, IL-2, IL-4, IL-6, IL-7, IL-8, IL-9, IP-10, MCP-1, MDC, and TNF-α (no details on time point)	IL-6, IL-8, and IL-10 were increased, but CSF tests for SARS-CoV-2 were negative in all patients.
Case report^171^	1. Contaminant COVID-19 infection and acute encephalopathy (*n* = 1) 2. Healthy control (*n* = 3)	CSF/plasma: multiplex cytokine assay (no details on time point)	CSF and plasma IL-6, IL-8, and IP-10 were increased compared to control, whereas MCP-1 was found only in CSF, but not in plasma.
Case report^107^	COVID-19-associated encephalitis (*n* = 2)	CSF: IL-1β, IL-6 (no details on time point)	CSF levels of IL-1β, IL-6, and ACE were increased.
Retrospective study^181^	Vietnamese adults: 1. Severe malaria (*n* = 62) 2. Control (*n* = 16)	CSF: S100B, NSE, tau protein (lumbar puncture was performed when clinically indicated as a routine part of the investigation after admission)	Mean concentration of tau, but not NSE and S100B, was significantly increased in patients with severe malaria vs. controls. Tau was associated with duration of coma, and S100B was associated with seizures.
Retrospective study^182^	Kenyan children with and without falciparum malaria (*n* = 143)	CSF: S100B, tau proteins (random samples of stored CSF, no details on time point)	Tau proteins were significantly elevated in children with cerebral malaria vs. malaria with prostration or malaria with seizures but normal consciousness, whereas S100B was associated with an increased risk of repeated seizures.
Observational cohort study^185^	Ugandan children with cerebral malaria: 1. Retinopathy-positive (*n* = 167) 2. Retinopathy-negative (*n* = 87)	CSF: tau protein, inflammatory protein markers (admission)	Tau, but not proinflammatory and anti-inflammatory cytokines and oxidative stress markers, was increased in retinopathy-positive vs. retinopathy-negative cerebral malaria.
Observational cohort study^186^	Ugandan children with cerebral malaria (*n* = 145)	CSF: tau (admission)	Tau was associated with neurological deficits at hospital discharge as well as with long-term neurological and cognitive deficits.
Post-mortem cohort study^183^	Pediatric populations: 1. Cerebral malaria (*n* = 9) 2. Severe malarial anemia (*n* = 5) 3. Non-malarial cause death (*n* = 5)	CSF/serum (36 biomarkers within 2–4 h of death): CRP, CXCL11 (I-TAC), eotaxin, Fas-L, FGF basic protein, G-CSF, GM-CSF, IFN-γ, IL-10, IL-12 (p70), IL-13, IL-15, IL-17, IL-1ra, IL-1β, IL-2, IL-4, IL-5, IL-6, IL-7, IL-8, IL-9, IP-10, MCP-1, MIP-1α, MIP-1β, MMP-9, PDGF-BB, RANTES, SDF-1α, sFas, sTNF-R1, sTNF-R2, TGF-beta1, TNF-α, and VEGF (post-mortem samples obtained within 2–4 h of death)	IP-10 was the only serum biomarker independently associated with CM mortality vs. SMA and NM. CSF IL-1ra, IL-8, IP-10, PDGF-BB, MIP-1β, Fas-L, sTNF-R1, and sTNF-R2 were significantly elevated in cerebral malaria vs. severe malarial anemia and non-malarial mortality group.
Observational cohort study^189^	1. Cerebral malaria survivors (*n* = 48) 2. Cerebral malaria, non-survivors (*n* = 12) 3. Mild malaria (*n* = 48) 4. Controls, healthy (*n* = 25)	Plasma (30 biomarkers): eotaxin, Fas-L, FGF basic protein, G-CSF, GM-CSF, IFN-γ, IL-10, IL-12 (p70), IL-13, IL-15, IL-17, IL-1ra, IL-1β, IL-2, IL-4, IL-5, IL-6, IL-8, IL-9, IP-10, MCP-1, MIP-1α, MIP-1β, PDGF-BB, RANTES, sFas, sTNF-R1, sTNF-R2, TNF-α, and VEGF (admission)	IP-10, sTNF-R2, and sFas were independently associated with increased risk of CM-associated mortality. The VEGF to IP-10, sTNF-R2, and sFas ratios distinguished cerebral malaria survivors from non-survivors.
Observational cohort study^184^	Children with *Plasmodium falciparum* infection: 1. Asymptomatic (*n* = 80) 2. Uncomplicated malaria (*n* = 69) 3. Severe malaria (*n* = 41) 4. Cerebral malaria (*n* = 22)	Plasma (biomarkers of the immune response): CX3CL1, MIG/CXCL9, neopterin, pentraxin 3, sCD14, sCD163, sTREM-1, and suPAR (mass-screen samples to detect parasite carriers)	Neopterin, suPAR, and fractalkine were strongly predictive of severe or cerebral malaria vs. uncomplicated malaria.
Observational cohort study^174^	Pediatric populations: 1. Acute miliary tuberculosis with secondary tubercular meningitis (*n* = 28) 2. Pure acute miliary tuberculosis (*n* = 25) 3. Suspected meningitis (*n* = 23)	CSF/serum: NSE, NPY, and S100B (no details on time point)	S100B, NSE, and NPY were significantly elevated in the acute miliary tuberculosis with secondary tubercular meningitis vs. other groups.
Observational cohort study^173^	1. Bacterial meningitis (*n* = 9) 2. Tuberculosis meningitis (*n* = 11) 3. Japanese encephalitis virus infection (*n* = 25) 4. Rickettsial infections (*n* = 21)	Plasma/CFS: albumin CSF: GFAP, NSE, S100B, and total tau (no details on time point)	GFAP and the albumin index were significantly higher in bacterial and tuberculosis meningitis groups, whereas total tau was elevated in the Japanese encephalitis virus infection group.
Observational cohort study^175^	1. Tuberculous meningitis (*n* = 44) 2. Controls: fatty filum (*n* = 11) 3. Pulmonary tuberculosis (*n* = 9)	CSF/serum (0–3 wk): GFAP, NSE, S100B, and multiple inflammatory markers (on admission and over 3 weeks)	S100B, NSE, GFAP, and inflammatory markers were elevated in CSF on admission and for up to 3 weeks, but not in serum, and were associated with CNS injury and/or outcomes.
Retrospective, observational cohort study^176^	1. HIV w/o antiretroviral treatment: 2. AIDS dementia complex (*n* = 55) 3. HIV with CNS opportunistic infections/tumors (*n* = 44) 4. HIV w/o neurological symptoms (*n* = 95) 5. Primary HIV (*n* = 16)	CSF: NfL (at the time of diagnosis, and within 1 week of initiating specific treatment)	NfL was associated with AIDS dementia complex.
Cross-sectional study^179,180^	1–7. HIV-infected subjects divided into 7 groups according to stage of the systemic disease, presence of overt HAD, and after ART (*n* = 121) 8. HIV-negative controls	Plasma and CSF: NfL (after informed consent of subjects)	Plasma and CSF NfL concentrations in the HAD group were significantly elevated compared to all other subgroups and in the neuroasymptomatic group with <50 CD4^+^ T-cell counts were significantly elevated compared to the other groups, except compared to the 50–199 CD4^+^ T-cell counts group and to the primary HIV infection group for CSF NfL only.
Observational cohort study^190^	HIV-infected persons: 1. ART <4 months after EDI (*n* = 9) 2. ART >14 months after EDI (*n* = 7)	Plasma and CSF: NfL, IL-6, MCP-1, sCD163, and TNF-α (first pair of samples at the evaluation in all participants, second pair of samples in subsets at 3 [*n* = 1] and 5 months [*n* = 1], and third sample 2 months thereafter [*n* = 1])	“Early ART” was significantly associated with lower CSF IL-6 levels, but not with other CSF or plasma markers, compared to “late ART.”
Retrospective, observational cohort study^191^	1. HIV-infected (*n* = 37) 2. HIV-negative controls (*n* = 54)	Plasma and CSF (in a subset of participants): NfL	No significant differences in plasma NfL between HIV-infected subjects and controls; statistically significant linear relationship between composite neuropsychological score (NPT-11) and plasma NfL in the univariate mixed-effect model
Randomized, active-controlled, multi-center, open-label, non-inferiority trial^192^	HIV-1-infected adults on elvitegravir (E)/cobicistat (C)/emtricitabine (F)/disoproxil fumarate (TDF) therapy at baseline 1. Switching to E/C/F/tenofovir alafenamide (TAF; *n* = 272) 2. Continuing E/C/F/TDF (*n* = 144)	Plasma (0, 24, and 84 wk): NfL (subsets of 37 HIV-positive adults: at baseline in subset [*n* = 34], second sample in subset of 11 from 16 HIV-positive participants with ART after median 28 weeks)	Although NfL levels in both groups were within the normal range, the NfL level in the E/C/F/TAF arm was significantly lower than in the E/C/F/TDF arm after 84 weeks of treatment.
Observational cohort study^178^	1. HIV (*n* = 67) 2. Control, HIV-negative (*n* = 45)	CSF: S100B, NfL (retrospective samples collected during 1999–2013)	S100B and NfL were increased in HIV vs. control, and NfL was associated with neurocognitive impairment.
Observational cohort study^177^	1. HIV with cognitive impairment (*n* = 10) 2. HIV with without cognitive impairment (*n* = 10)	CSF: untargeted proteomics by mass spectrometry (retrospective samples collected during 1998–2013)	Subjects with HIV-associated neurocognitive disorders had higher abundance of CSF extracellular vesicles and proteins related to synapses, glial cells (incl., GFAP, S100B), inflammation, and stress responses vs. patients without cognitive impairment.

AIDS, acquired immunodeficiency syndrome; ART, antiretroviral therapy; CD, cluster of differentiation; COVID-19, coronavirus disease 2019; CSF, cerebrospinal fluid; CX3CL1, fractalkine, interferon-inducible T-cell α chemoattractant; EDI, estimated date of infection; Fas-L, Fas-ligand; FGF, fibroblast growth factor; G-CSF, granulocyte colony-stimulating factor; GFAP, glial fibrillary acidic protein; GM-CSF, granulocyte-macrophage colony-stimulating factor; HAD, HIV-associated dementia; IFN-γ, interferon-γ; IL, interleukin; IP-10, interferon-γ-induced protein-10; MCP-1, monocyte chemoattractant protein-1; MDC, macrophage-derived chemokine; MIG/CXCL9, monokine induced by γ-interferon/chemokine (C-X-C motif) ligand 9; MIP, macrophage inflammatory protein; MMP-9, matrix metallopeptidase 9; NfL, neurofilament light; NPY, neuropeptide Y; NSE, neuron-specific enolase; PDGF-BB, platelet-derived growth factor; pNfH, phosphorylated neurofilament heavy chain protein; RANTES, regulated on activation, normal T cell expressed and secreted chemokine; S100B, S100 calcium-binding protein B; sCD, soluble cluster of differentiation; SDF-1α, stromal cell-derived factor 1α; sFas, soluble Fas; sTNF-R, soluble TNF receptors; sTREM-1, soluble triggering receptor expressed by myeloid cells 1; suPAR, soluble urokinase-type plasminogen activator receptor; TNF-α, tumor necrosis factor α; VEGF, vascular endothelial growth factor.

Protein biomarkers of CNS damage have also been detected after a variety of acute injury modalities.^193–201^ Elevations of brain injury biomarkers, including GFAP and ubiquitin C-terminal hydrolase L1 (UCH-L1), have been detected in humans and animals acutely after diverse brain injuries, including TBI,^202,203^ ischemic/hemorrhagic stroke,^198^ cardiac arrest,^204^ hypoxia,^196^ seizures,^194^ and even drug toxicity.^193^ Consistent with these observations, high levels of NSE, S100B, GFAP, and other biomarkers (e.g., neurofilament proteins) can be used to support the prognosis of poor neurological outcome after cardiac arrest, as recommended by the American Heart Association (AHA) Guidelines, and in the recent AHA report on standards for prognostication; serial testing is recommended.^197,201^ A recent study of 717 patients reported that UCH-L1 and GFAP were optimal BB predictors of outcome as early as 24 h after cardiac arrest.^205^ Use of BBs as an adjunct to other diagnostic modalities was recommended. Monitoring of plasma concentrations of GFAP and other brain injury biomarkers, including S100B and NSE, has also been shown to detect brain injury in children on extracorporeal membrane oxygenation (ECMO).^206, 207^

Interestingly, serial serum S100B sample analyses demonstrated significant increases in biomarker levels and increasing trajectory in adult ECMO patients developing intracranial lesions.^208^ Serial NSE levels were associated with neurological outcomes after cardiopulmonary resuscitation in patients on ECMO.^209^ Increased blood levels of NSE and S100B were associated with mortality and poor neurological outcomes in adult patients with accidental hypothermia treated with rewarming and/or extracorporeal life support, including ECMO.^210^

BBs are also altered in AD patients. GFAP measured in serum is increased in AD and correlates with cognitive decline.^211^ Temporal profiles of serum NfL levels are associated with cognitive decline^212^ and can predict clinical progression even in pre-symptomatic AD.^213^ At present, levels of BBs of brain injury in COVID-19 patents are unknown. However, recent studies have shown that protein biomarker assays can detect even low levels of UCH-L1, S100B, and GFAP elevated after sport concussion not associated with CNS pathology detectable by computed tomography (CT) scans.^214,215^

BBs have yet to be fully exploited for medical management of brain injury. However, as documented in Table 3, research laid the foundation for U.S. Food and Drug Administration (FDA) accelerated approval of measures of UCH-L1 and GFAP for acute diagnosis of TBI not associated with CT abnormalities versus moderate TBI associated with CT abnormalities. This clearance was provided under the FDA's Break-through Devices Program.^216–218^ Studies of BBs potentially assessing brain injury in COVID-19 patients could explore the BBs' previously reported properties, including: quantifying injury magnitude; aiding in outcome prediction; defining the presence of diffuse injury versus mass lesions; assessing injury to different cell types (e.g., neurons vs. glia); assessing subcellular loci of injury (e.g., cell body, axons, myelin sheath, and pre- and post-synaptic structures); tracking contributions from different injury mechanisms and their time courses (e.g., necrosis vs. apoptosis); and detecting secondary insults during patient care.^198,202,203,219–221^

**Table 3.** Human Studies of Brain Injury BBs in CNS Disorders and Disorders Associated with Neurological Complications

Study design	Disorder groups (patient nos.)	Biomarkers (time points)	Major findings
Prospective cohort study^233^	1. Ischemic stroke (*n* = 26) 2. Control (*n* = 26)	Serum S100B (1–10 d)	S100B was elevated in ischemic stroke compared to control and correlated with neurological deficits, infarction, and edema, but not with the functional prognosis.
Prospective cohort study^234^	1. Ischemic stroke (*n* = 21) 2. TBI (*n* = 10) 3. TIA (*n* = 18) 4. Control (*n* = 28)	Serum S100B (0–24 h)	S100B correlated with neurological deficits in ischemic stroke and TBI; S100B temporal profile in ischemic stroke was different compared to TIA or TBI.
Prospective cohort study^235^	Ischemic stroke (*n* = 32)	Blood S100B, NSE (6–120 h)	S100B and NSE correlated with the neurological deficit and the final infarct volume; S100B concentrations were associated with the functional outcome and indicated a poor functional 3-month outcome.
Prospective cohort study^236^	1. Ischemic stroke (*n* = 93) 2. ICH (*n* = 42)	Serum GFAP (6 h)	GFAP allowed to detect ICH in acute stroke.
Retrospective study (samples from a NINDS r-tPA trial)^237^	Ischemic stroke groups: 1. r-tPA (*n* = 178) 2. Placebo (*n* = 181)	Serum S100B, NSE, and MBP (0–24 h)	S100B, NSE, and MBP levels were associated with CT lesion volume and outcomes, but did not reveal differences between r-tPA and placebo groups.
Prospective cohort study^238^	Ischemic stroke (*n* = 66)	Blood NSE, and tau (3–120 h)	NSE was associated with the neurovascular status on admission and correlated with the functional 3-month outcome, whereas tau was correlated with neurological deficits and infarct volume, but not with the neurovascular status on admission.
Prospective cohort study^239^	1. Ischemic stroke (*n* = 45) 2. ICH (*n* = 18)	Serum GFAP (1–48 h)	GFAP was correlated with ICH volume that allowed to differentiate between ICH and ischemic stroke.
Prospective, multi-center cohort study^240^	1. Ischemic stroke (*n* = 83) 2. ICH (*n* = 14)	Blood GFAP, S100B, and NSE (0–24 h)	GFAP, but not S100B or NSE, allowed to differentiate between ICH and ischemic stroke.
Retrospective, single-center analysis^241^	CA (*n* = 44)	Serum GFAP (12, 24, and 48 h)	GFAP was significantly higher in patients with a poor outcome at 12 and 24 h without TH and at 48 h with TH. GFAP was a specific predictor of poor 6-month neurological outcome with or without TH treatment.
Prospective, randomized controlled trial^242^	1. TBI (severe): (*n* = 65) 2. Healthy control (*n* = 25)	Plasma sE-selectins, sVCAM-1, sICAM-1, TNF-α, and IL-10 (0, 12, 24, and 48 h)	Concentrations of sVCAM-1, sE-selectin, TNF-α, and IL-10 were correlated with hypertonic fluid treatment.
Prospective, observational cohort study^206^	1. Neonatal/pediatric patients on ECMO (*n* = 22) 2. Control, healthy (*n* = 99) 3. Control, neonatal ICU patients without neurological injury (*n* = 59)	Plasma GFAP (6, 12, and every 24 h after cannulation)	GFAP was associated with brain injury and mortality.
Prospective cohort study^243^	1. Ischemic stroke (*n* = 56) 2. Control (crural varices; *n* = 38)	Serum S100B, tau protein (1–10 d)	S100B was detected in all patients and in 55% of controls. Acute tau protein concentrations neither correlated with neurological deficits nor disability.
Randomized controlled trial^244^	Ischemic stroke groups: 1. EPO (*n* = 76) 2. Placebo (*n* = 87)	Serum GFAP, UCH-L1, and S100B (1–7 d)	GFAP, UCH-L1, or S100B alone were corrected with NIHSS before drug treatment, whereas a significant difference between EPO vs. placebo group was observed only for UCH-L1.
Pilot study^245^	Unconscious resuscitated CA patients treated with mild hypothermia (*n* = 22)	Serum tau (2, 6, 12, 24, 48, and 96 h after CA)	High tau at 48 and 96 h was associated with poor 6-month neurological outcome after CA.
Observational cohort study^194^	1. Epileptic seizure (*n* = 52) 2. Control (*n* = 19)	CSF/plasma UCH-L1 (48 h)	CSF and plasma UCH-L1 were correlated with age in seizure, but not in control, patients. Plasma UCH-L1 was significantly higher in patients after recurrent seizures.
Prospective cohort study^246^	1. Ischemic stroke (*n* = 150) 2. Control (*n* = 101)	Serum NSE (72 h)	NSE was correlated with stroke severity and disability.
Interventional, randomized, open label (phase 3)^247^	Pediatric mild TBI (*n* = 446)	Serum S100B (3 h)	The study suggested that early S100B determination in children with mild TBI could potentially reduce the number of CT scans and save hospitalization costs.
Prospective, multi-center cohort study^248^	1. Ischemic stroke (*n* = 163) 2. ICH (*n* = 39) 3. Control (stroke mimic; *n* = 3)	Plasma GFAP (4.5 h) plasma	GFAP allowed to differentiate ICH from ischemic stroke and stroke mimic.
Prospective cohort study^249^	1. Ischemic stroke (*n* = 61) 2. ICH (*n* = 79) 3. Control (high risk; *n* = 79)	Serum S100B, NSE (12–48 h)	S100B and NSE were elevated in stroke and correlated with selected functional outcomes at 60 days.
Prospective cohort study^250^	ICH (*n* = 176)	Serum tau protein (6 h)	Tau allowed to predict 3-month poor outcome.
Prospective cohort study^251^	1. Ischemic stroke (*n* = 776) 2. ICH (*n* = 139)	Plasma S100B, RAGE (24 h)	S100B was significantly increased in ischemic stroke vs. ICH.
Retrospective, observational study^252^	CA (*n* = 25)	Serum total tau (serial blood samples during the first 5 days after resuscitation)	Tau kinetics was associated with 6-month outcome.
Multi-center, prospective^253^	TBI (mild, moderate, and severe; *n* = 215)	Plasma GFAP-BDP (24 h)	GFAP-BDP allowed to detect the presence and severity of abnormal CT findings.
Double-blind, randomized clinical trial^254^	TBI (moderate to severe) groups: 1. Rosuvastatin (*n* = 19) 2. Placebo (*n* = 17)	Plasma TNFα, IL-1β, IL-6, and IL-10 (72 h)	TNF-α, but not IL-1β, IL-6, and IL-10, was significantly different in treatment group vs. placebo.
Prospective cohort study^255^	1. Ischemic stroke (*n* = 100) 2. Control (*n* = 101)	Serum NSE, IL-10 (72 h)	NSE and IL-10 were increased in stroke vs. control and correlated with neurological deficits.
Prospective cohort study^256^	Ischemic stroke (*n* = 75)	Serum NSE (24 h)	NSE was correlated with infarct volume detected by CT scan, GCS, and 30-day neurological outcomes.
Multi-center, prospective cohort study^257^	1. TBI (*n* = 206) 2. Control (*n* = 175)	Plasma/serum GFAP, UCH-L1 (24 h)	GFAP and/or UCH-L1 allowed to discriminate between TBI patients with and without intracranial lesions on CT scan and correlated with functional 3-month outcomes.
Observational cohort study^196^	1. HIE neonates (*n* = 16) 2. Control (*n* = 11)	Serum GFAP, UCH-L1 (cord blood)	UCH-L1 was elevated in HIE neonates and associated with cortical injury and later motor and cognitive developmental outcomes, whereas the GFAP temporal profile predicted injury to the basal ganglia and white matter and motor developmental outcomes.
Prospective cohort study^258^	Ischemic stroke (*n* = 83)	Blood NSE (24 h)	Specific NSE temporal profile was correlated with atrial fibrillation and hemorrhagic transformation.
Prospective cohort study^259^	1. Ischemic stroke (*n* = 88) 2. ICH (*n* = 32) 3. Control (*n* = 32)	Serum NSE, CRP (72 h)	NSE and CRP were increased in acute stroke compared to control, but were not statistically different between ischemic stroke and ICH.
Prospective, single-center pilot study^260^	1. Ischemic stroke (*n* = 31) 2. ICH (*n* = 12) 3. Vertigo (nonvascular; *n* = 22) 4. Control (*n* = 15)	Serum GFAP, S100B (24 h)	Serum GFAP was not significantly different between groups, whereas S100B was significantly higher in stroke patients.
Prospective, randomized, double-blinded clinical trial^261^	TBI (severe) groups: 1. Epoprostenol (*n* = 23) 2. Placebo (*n* = 23)	IL-6, IL-8, sICAM-1, CRP, and ADMA (1–5 d)	IL-6 and CRP, but not IL-8 or sICAM-1, were significantly different in treatment group vs. placebo.
Prospective cohort study^262^	1. ICH (*n* = 35) 2. Control (*n* = 32)	Blood S100B, NSE, and HSP70 (24 h, 5 d)	S100B and NSE were increased in ICH compared to control; S100B was correlated with NIHSS, bleeding volume, and GCS.
Prospective cohort study^263^	1. Ischemic stroke (*n* = 34) 2. Control (*n* = 34)	MBP, IMA (12 h) Serum	No statistically significant differences between stroke and control groups; statistically significant correlations with NIHSS score (*p* = 0.002; *r* = 0.43) and IMA (*p* = 0.015; *r* = 0.344) levels
Prospective, observational study^264^	1. Ischemic stroke (*n* = 94) 2. ICH (*n* = 35) 3. TIA (*n* = 13) 4. Control (*n* = 40)	Serum S100B (48 h)	S100B was elevated in stroke groups compared to TIA and control.
Prospective, observational study^207^	Children <18 years groups: 1. ECMO for any indication (*n* = 80) 2. Control (critically ill patients admitted to the PICU during the same study period who did not require ECMO; *n* = 28)	Plasma GFAP, CCL2, NSE, S100B, and BDNF	GFAP, CCL2, NSE, and S100B were significantly higher in patients with unfavorable outcome than in those with favorable outcome. GFAP and ICAM-5 were elevated in patients with vs. without abnormal neuroimaging findings.
Retrospective cohort study^265^	1. Ischemic stroke (*n* = 36) 2. ICH (*n* = 10)	Plasma GFAP, RPB4 (6 h)	GFAP was significantly higher in ICH vs. ischemic stroke.
Prospective study^266^	Ischemic stroke (r-tPA; *n* = 67)	Serum NSE (24 h)	NSE was correlated with NIHSS score.
Multi-center, prospective cohort study^267^	TBI (mild, moderate, and severe; *n* = 215)	Serum GFAP-BDP	GFAP-BDP had the ability to predict and discriminate injury severity detected with CT scan.
Interventional, randomized, prospective, double blind (phase 1)^268^	TBI (severe; *n* = 48)	S100B, NSE (1–5 d)	Combination of S-100B and Morris-Marshall improved the prediction of clinical outcome; S100B was associated with ICP and CPP and discriminate brain injury detected by CT scan, whereas NSE was associated with mortality.
Prospective cohort study^269^	1. Ischemic stroke (*n* = 49) 2. ICH (*n* = 23) 3. Control (*n* = 52)	Serum GFAP, NR2 antibodies (12–72 h, 1 and 2 wk)	Combination of GFAP and NR2 antibodies discriminated ischemic stroke vs. ICH.
Prospective two-center study^270^	TBI (*n* = 324)	Serum GFAP, UCH-L1 (0, 1, 2, 3, and 7 d)	GFAP was associated with levels of recovery and favorable acute outcomes and mortality, whereas UCH-L1 was not able to predict the outcome in the multi-variate logistic regression model.
Prospective, multi-center observational study^271^	TBI (mild and moderate; *n* = 273)	Serum GFAP, UCH-L1, and S100B (6 h)	UCHL1 and the combination of GFAP and UCH-L1 were sensitive for a positive CT scan.
Prospective cohort study^272^	1. Ischemic stroke (*n* = 65) 2. ICH (*n* = 43)	Serum GFAP (2–6 h)	GFAP was able to differentiate ICH vs. ischemic stroke.
Interventional, single-center, randomized, blinded controlled (phase 2)^273,274^	DAI groups^273^: 1. Progesterone (*n* = 24) 2. Control (*n* = 24)	Serum S100B, IL-1β, IL-6, TGF-1β, and MDA (24 h and 6 d)	Serum S100B, IL-1β, IL-6, TGF-1β, and MDA were significantly elevated in DAI patients and correlated with treatment vs. control.
	DAI groups^274^: 1. Progesterone (*n* = 16) 2. Control (*n* = 16)	Serum NSE, ICAM-1 (24 h and 6 d)	Serum ICAM-1 was decreased in the progesterone group, whereas there were no significant differences in serum NSE between the study groups.
Prospective, two-center cohort study^275^	1. TBI (*n* = 324) 2. Control (orthopedic trauma; *n* = 81)	Plasma GFAP, UCH-L1 6 h	GFAP levels correlated with initial severity of TBI (GCS).
Randomized, double-blind, placebo controlled (phase 3)^276^	1. Complicated mild TBI (*n* = 34) 2. Control (*n* = 19)	Plasma GFAP, tau protein, and amyloid β (0–24 h, 30 d, and 90 d)	GFAP, total tau, and amyloid β were increased up to 90 days after TBI compared to control; amyloid β was correlated with clinical outcome, whereas total tau was correlated with clinical and radiological TBI severity.
Prospective, clinical biobank study of data from the randomized Target Temperature Management After Cardiac Arrest trial, an international, multi-center study with 29 participating sites.^205,277–280^	CA (*n* = 686)^277^	Serum NSE (24, 48, and 72 h after CA)	High, serial NSE were strong predictors of poor 6-month neurological outcome after CA. Targeted temperature management at 33°C or 36°C did not significantly affect NSE levels.
	CA (*n* = 689)^278^	Serum tau, NSE (24, 48, and 72 h after CA)	Increased tau was associated with poor outcome at 6 months after CA. Tau demonstrated better prognostic value compared to NSE.
	CA (*n* = 687)^279^	Serum S100B (24, 48, and 72 h after CA)	High S100 was predictive of poor 6-month outcome, but did not add value to present prognostication models with or without NSE.
	CA (*n* = 717)^280^	Serum NfL (24, 48, and 72 h after CA)	NfL levels had significantly greater performance than the other serum markers (i.e., tau, NSE, and S100). NfL at 24 h after CA is a highly predictive marker of poor 6-month neurological outcome.
	CA (*n* = 717)^205^	Serum GFAP, UCH-L1, and NSE (24, 48, and 72 h)	GFAP and UCH-L1 discriminated between good and poor 6-month neurological outcome when used alone or in combination. The combined model was superior to GFAP and UCH-L1 separately and NSE at all time points.
Interventional, prospective, randomized, not blinded^281^	TBI groups: 1. Memantine (*n* = 22) 2. Control (*n* = 19)	Serum NSE (7 d)	Serum NSE was significantly different in treatment group vs. control and correlated with GCS.
Multi-center, prospective cohort study^282^	TBI (*n* = 217)	Plasma total tau, hyperphosphorylated tau (<24 h and 44.5 d)	Plasma hyperphosphorylated tau levels and hyperphosphorylated tau/total tau ratio were associated with neurological outcomes.
Prospective, observational cohort study^215^	1. Sport-related concussion (*n* = 36) 2. Baseline (*n* = 110)	Serum GFAP, UCH-L1, total tau, and NfL (baseline and after sport-related concussion)	GFAP, total tau, and NfL were increased after sport-related concussion vs. baseline.
Prospective, observational cohort study^214^	Baseline and after concussion: 1. Injured football players (*n* = 106) 2. Matched uninjured football players (*n* = 84) 3. Non-contact-sport athletes (*n* = 50)	Serum GFAP, UCH-L1, S100B, SBDP150, IL-6, IL-1 receptor antagonist (0–6, 24, 48 h)	UCH-L1, S100B, SBDP150, IL-6, and IL-1 receptor antagonist were significantly elevated at the early acute phase post-injury vs. baseline, whereas GFAP was elevated post-injury vs. baseline in concussed athletes with a loss of consciousness or amnesia; IL-1 receptor antagonist was correlated with recovery.

BDNF, brain-derived neurotrophic factor; CA, cardiac arrest; CCL2, monocyte chemoattractant protein 1/chemokine (C-C motif) ligand 2; CPP, cerebral perfusion pressure; DAI, diffuse axonal injury; ECMO, extracorporeal membrane oxygenation; EPO, erythropoietin; GCS, Glasgow Coma Scale/Score; GFAP, glial fibrillary acidic protein; GFAP-BDP, GFAP breakdown products; HIE, hypoxic-ischemic encephalopathy; ICAM, intercellular adhesion molecule; ICH, intracerebral hemorrhage; ICP, intracranial pressure; IL, interleukin; MDA, malondialdehyde; NIHSS, National Institutes of Health Stroke Scale/Score; NR2, N-methyl-d-aspartate receptor subunit 2; PICU, pediatric intensive care unit; RPB4, DNA-directed RNA polymerase II subunit B4; S100B, S100 calcium-binding protein B; SBDP150, αII-spectrin breakdown product 150 KDa; TGF-1β, transforming growth factor 1β; TH, therapeutic hypothermia; TIA, transient ischemic attack; TNF-α, tumor necrosis factor α; tPA, tissue plasminogen activator; VCAM-1, vascular cell adhesion molecule 1.

Although the clinical utility of BBs for detection of CNS injury is widely recognized, the influence of mechanisms regulating transport of molecules into and out of the brain is poorly understood. Moreover, the literature is often compromised by the assumption that the same processes underlying movement of molecules from the blood to the brain, commonly referred to the blood–brain barrier (BBB), also underlie movement of molecules from the brain to the blood, that is, the BBB, as would be the case for BBs originating in the brain and detected in blood. Some studies further assume that S100B can be a marker of BBB opening even though S100B is also found in peripheral tissue (e.g., a previous work^222^). More generally, investigations have failed to appreciate the complexity and dynamic qualities of movement of substances between CNS and extra CNS compartments, as highlighted by descriptions of the glymphatic system involved in the removal of molecules from interstitial spaces of the brain.^223–225^ BBB opening after acute injuries such as TBI are biphasic and exhibit acute and chronic phases with different characteristics and mechanisms.^226^

A dual role for astrocytes at the BBB has been documented in studies of CNS injury. Astrocytes can mediate either increases or decreases in BBB permeability depending upon the type of injury (for a review, see a previous work^227^). Even exercise and stress have been reported to open the BBB.^228–230^ A comparison of levels of GFAP and UCH-L1 in rats after experimental TBI found differing levels of the two BBs in CSF versus blood.^231^ These data suggest that levels of BBs may result from different transport and/or clearance processes that need to be examined individually for each marker. Similar conclusions were drawn for NSE and S100B in studies conducted in severe TBI patients.^232^ The prominent pathology observed in the cerebrovasculature of COVID-19 patients supports the possibility of disruptions of the normal processes regulating exit and entry of molecules into and out of the brain, and studies of these disturbances present promising areas of investigation. As has been done successfully in the past, this research can occur in parallel with studies examining the clinical utility of BBs in COVID-19 patients.

Although not widely appreciated, TBI, stroke and other “acute” brain injuries initiate progressive and evolving pathological events. Similarly, infection by the SARS-CoV-2 virus can initiate pathologies attributable to both the initial insult and secondary injures that can continue for as yet undetermined durations. This progressive pathology is confounded by sustained infection in COVID-19 patients. Thus, we have pointed out the need for serial measurements of BBs in clinical studies given that differing biokinetics of individual markers will produce useful data relevant to understanding the durations of different pathological responses to SARS-CoV-2 and disturbances in processes regulating compartmentalization of molecules originating in the brain.

## Integration of Central Nervous System Injury Diagnostic Tools

As in every area of medicine, BBs of brain injury are optimally used in conjunction with other diagnostic tools and can synergistically improve the accuracy and utility of other diagnostic modalities.

### Brain imaging

Significant CNS neuroimaging findings have been reported after SARS-CoV-2 infection, including cerebrovascular-related injury, meningitis, and encephalitis (see Table 1). Some CNS abnormalities detected by imaging may be related to intracranial cytokine storms, which may result in BBB breakdown without direct viral invasion or parainfectious demyelination.^143^ It will be important to characterize the optimal MRI and CT imaging protocols to detect CNS damage in neurologically symptomatic patients, including emphasis on protocols best suited to detect even subtle microvascular damage. These findings and any changes in them over time will be useful in determining the validity of the BB results.

### Neurological assessments

Common Data Elements (CDEs) have been created for a number of neurological disorders, including TBI, stroke, and neuromuscular diseases (https://www.commondataelements.ninds.nih.gov/). These CDEs comprehensively embrace domains including neurological assessments, imaging, and laboratory assessments. Existing CDEs can readily be adapted to COVID-19 studies. The Neurocritical Care Society (https://www.neurocriticalcare.org/home) has organized a multi-center, international collaborative effort to develop guidelines and tools to align data and biological sample collection protocols, to facilitate their integration into master datasets.

### Genotyping

As has been the case for TBI and stroke, knowledge of individual genotypes may improve the prediction of differing clinical outcomes of COVID-19 patients.^283–286^ Although individual, non-genetic susceptibility may influence the occurrence or severity of brain injury caused by SARS-Cov-2, variation in susceptibility to SARS-Cov-2 may also be attributable to individual differences in genetic susceptibility factors in infected individuals. Susceptible persons may have impaired innate abilities to fight viruses attributable to suboptimal interferon responses^287^ or inability to mount adequate cell-mediated immune responses.^288^ Mutations in Toll-like receptor 3 (TLR3) have been tied to increased herpes simplex encephalitis in patients,^289^ suggesting that immune system impairment can increase viruses' abilities to invade the brain and cause injury. Polymorphisms of the sulfonylurea receptor 1-transient receptor potential melastatin 4 (Sur1-Trpm4) cation channel are also associated with the development of malignant cerebral edema after acute brain injury.^290,291^ Importantly, investigators have recently identified a 3p21.31 gene cluster as a genetic susceptibility locus in patients with COVID-19 with respiratory failure and confirmed a potential involvement of the ABO blood-group system.^292,293^

Other studies have reported associations between blood-group types and disease severity and multi-organ dysfunction in COVID-19.^294,295^ In the first publications of the COVID Human Genetic Effort, researchers found that 101 of 987 patients with life-threatening COVID-19 pneumonia had neutralizing immunoglobulin G auto antibodies against the interferons (IFNs), including IFN-ω and 13 types of IFN-α or both, at the onset of critical disease. Some patients also had autoantibodies against the other three type I IFNs. Ninety-five percent of these patients were male. These autoantibodies also neutralized corresponding type I IFNs to block SARS-CoV-2 infection *in vitro*.^296^

Other investigators discovered that among nearly 660 persons with severe COVID-19, >3.5% were completely missing a functioning gene. Further experiments showed that immune cells from those 3.5% did not produce any detectable type I IFNs in response to SARS-CoV-2.^297^ Consequently, both groups lack effective immune responses that depend on type I IFNs. Whether IFNs were neutralized by autoantibodies, produced in insufficient amounts because of genetic influence, or because IFNs induced an inadequate antiviral response, their IFN deficits seem to be a commonality among a subgroup of people who suffer from severe COVID-19 pneumonia. It will be important to determine whether the same IFN deficits contribute to enhanced CNS injury in COVID-19 patients.

Among the known genetic risk factors for CNS injury, the potential contribution of the apolipoprotein E (ApoE4) genotype should be determined, especially given that cerebrovascular damage may be a prominent characteristic of brain injury in COVID-19 patients. ApoE4 is associated with increased risk of intracerebral hemorrhage (ICH)^298^ and predicts unfavorable neurological outcome after TBI and stroke.^283,299^ ApoE4 also appears to have direct effects on the cerebrovascular system and may affect neurovascular functions independently of its known influence on amyloid beta (Aβ) pathology in AD.^300,301^

### Use of health information technology to support integration

Unlike past pandemics, the fight against COVID-19 can utilize recent advancements in health information technology (HIT). Barriers to research during pandemics, such as those requiring in-person contact (e.g., obtaining informed consent and acquiring CDEs), can be more readily obtained by utilizing HIT. Electronic health record (EHR)-embedded strategies for CDE collection and customized computable composite outcomes can be gathered and deidentified while patients are contagious, in support of, although not replacing, traditional approaches. EHR-embedded strategies can be linked to clinical laboratories for remnant serum/plasma sample collection and storage, reducing the need for dedicated blood draws and further minimizing exposure of nurses and/or healthcare personnel to potential infection. EHR-embedded data also lends itself to machine learning or artificial intelligence approaches.

Multi-center databases of patients with COVID-19 built with CDEs mapped to interoperable data standards, such as the Observational Medical Outcomes Partnership (OMOP) Common Data Model,^302,303^ could be used to develop predictive models for biodigital identification of patients at risk. More important, these approaches may also identify modifiable risk factors, susceptibility risk factors in patients, and aid in validation of best practices to inform patient care and future clinical trials.

## Clinical Applications of Blood Biomarkers to Improve Medical Management of COVID-19 Patients

The identification of applications of BBs that could improve medical practice for management of CNS injuries in COVID-19 patients is a prerequisite for design of research studies assessing their clinical utility. Unlike single brain injuries such as stroke or TBI, COVID-19 is an evolving disease process that will require serial BB assessments to provide optimal data. In addition, BBs for different brain injury processes may have different expression profiles,^304–307^ an observation that may provide additional diagnostic information.

We have reviewed evidence for the neuroinvasive capacity of SARS-CoV2 and potential consequence of direct neuronal cell by SARS-CoV-2. In light of increasing evidence for CNS infection as well as for ischemic damage, microinfarcts, and vascular pathology, there is a clear medical need for diagnostic tests that potentially aid with screening and identification patients, improve patient management of those with CNS involvement, and prediction of short- and long-term neurological consequences. The potential for a blood-based test for neurological injury may help clinicians identify patients early in the disease process and assist in determining the need for advanced imaging and initiation of current or potential new therapies.

An accurate diagnostic test that could reflect the degree of ongoing, worsening, or improving neurological involvement would aid clinicians in following patients' response to therapy and their overall clinical course. These tests may be particularly useful when patients are in critical condition, on ventilators, sedated, and possibly with chemical paralysis for which movement to imaging suites and clinical evaluation of neurological functions are difficult. These patients are especially vulnerable to secondary CNS injuries, such as hypoxia, frequently observed during the clinical course of the disease. Finally, evidence of CNS involvement may portend long-term neurological consequences. Thus, BBs could help clinicians determine the potential for longer-term or delayed neurological deficits and disease and direct patients to appropriate rehabilitation regimens.

As pointed out in the following section, clinical applications of BB diagnostics would primarily affect medical practice in acute and critical care environments. Important related research applications include assessments of risks for neurodegenerative diseases and design of clinical trials to treat COVID-19.

### Screening

Most screening will occur in emergency rooms and acute care facilities. Prolonged, potentially chronic systemic pathologies resulting from SARS-CoV-2 infection are of increasing concern, including recent reports of acutely undetected cardiovascular pathologies such as myocarditis.^295^ Given the distribution of ACE2 receptors in the brain as well as systemic organs, it may be prudent to screen all COVID-19 patients for occult brain injuries that might, as in myocarditis for cardiac disease, later become clinically expressed as persistent neurological deficits and/or increased risk for neurodegenerative diseases (see sections above). In addition, elevations of brain injury biomarkers may be sensitive indicators to alert clinicians to accompanying injuries to systemic organs and the need to screen accordingly (e.g., a troponin test for cardiovascular damage). Conversely, lower levels of BBs may predict a more favorable neurological clinical course.

Non-restrictive screening studies can be used to identify variables that could influence BB levels in individual patients. Unless incorporated into predictive models, these variables could compromise the predictive value of BBs. A more restricted screening strategy should be based on the most current literature and minimally select patients on the basis of risk criteria, including disease severity, the presence of altered mental status and other acute neurological symptoms, previous histories of CNS injuries, including stroke and TBI, evidence of hypoxia, smoking,^308^ dementia,^16, 309–311^ and the presence of any other pre-existing medical conditions that may render them more vulnerable to disease progression. Age is a major factor affecting disease severity and outcome of COVID-19 patients^31,310,312^ (also see a previous work,^313^ an observation which may require the development of age-adjusted norms for biomarkers such as GFAP and UCH-L1^314^). Finally, in light of recent data on genetic influences on COVID-19 disease progression (see above section), genetic screening is advisable.

### Acute diagnosis and triage

As reviewed in an earlier section, the potential utility of the BBs, GFAP and UCH-L1, to acutely diagnose even low levels of CNS injury, such as sport concussion,^214,215^ and to triage moderate and severe TBI patients for imaging has been demonstrated.^217,271,315,316^ In addition, BBs in combination with refined imaging classification systems can also assist in assessments of acute patient care needs (e.g., discharge vs. hospitalization), thereby assisting in maintaining a clinical care pathway that optimizes management of potentially scarce medical resources.^313,317^

### Prognosis

As pointed out above for screening tests, **i**dentifying or ruling out neurological involvement using BB measurements could provide prognostic information, especially with regard to early prediction of disease progression. This is the case for other BBs, such as D-dimer, fibrinogen, and troponin, in COVID-19 patients.^318–323^ Troponin elevation has been shown to also specifically identify COVID-19 patients who develop cardiovascular complications,^323^ supporting the need to determine whether elevated neuronal BBs could also be an early indicator in those patients who are developing neurovascular complications such as stroke and microvascular damage. Serial assessments of BBs could be important to identify early and continuing biomarker elevations that could trigger more frequent neurochecks in hospitalized patients and potentially establish who will benefit from neurocognitive rehabilitation in hospital settings. Investigators have examined the potential for symptom clusters in COVID-19 patients for prediction of injury severity associated with the need for respiratory support.

The duration of confusion was longer in more severe clusters whereas loss of smell or taste was reported over a longer duration in milder clusters, suggesting that CNS injury increased in more severely affected COVID-19 patients.^324^ It will also be important to identify potential confounders of accurate prognosis, and data provided by screening studies can provide assist in characterizing variables influencing disease severity and outcome. A useful model may be the International Mission for Prognosis and Analysis of Clinical Trials project, a study of outcome predictors in severe TBI patients.^325–327^

### Management of therapeutic interventions in hospitalized patients

There is growing recognition by critical care specialists that all critically ill patients must be concurrently managed for risks both to systemic organs and to the brain.^328^ COVID-19, as a systemic disease with potentially severe CNS consequences, confirms the merits of this approach. In addition to risks for CNS injury resulting directly from the virus, patients can suffer brain injuries as a result of secondary insults, such as hypoxia or hypotension, experienced during their hospital stays. A brain infected by the SARS-CoV-2 virus may be more vulnerable to secondary insults than an uninfected brain, given that the CNS has a low tolerance for repeated injuries.^329–331^ As emphasized in the section below, aside from limited information provided by imaging and surface electroencephalography measurements, there are no available tools to assess directly the evolution of brain injury in hospitalized patients or in ICUs.

Such information is essential in order to provide timely guidance in management strategies seeking to attenuate brain injury such as improved CNS oxygenation and perfusion as well as reductions in intracranial pressure. For example, BBs of brain injury and inflammation could be used to detect acute brain injury in a patient with COVID-19 with an accompanying “cytokine storm” who develops severe acute lung injury (ALI). Severe ALI mandates use of a protective lung strategy with tolerance of marginal oxygen saturation. Elevations of levels of brain injury biomarkers would also raise concerns over hypoxic brain injury from systemic hypoxemia or stroke, or possibly from direct CNS involvement of COVID-19, prompting an increase in FiO_2_ and an emergency diagnostic workup for stroke. Thus, early identification of acute brain injury complicating COVID-19 using serum biomarkers could prompt early interventions and improve outcomes.

### Clinical applications in acute care facilities and emergency rooms

Existing FDA approval of use of BBs for acute diagnosis of mild versus moderate TBI^217^ and numerous publications on the use of BBs for acute diagnosis of brain injury^220,221^ strongly support the feasibility of studies of BBs for acute detection of CNS involvement. Diagnostic tools that can assist in rapid diagnosis of potential CNS injury from SARS-CoV-2 infection are badly needed in order to inform appropriate medical care and establish evidence-based clinical pathways. Although altered mental status is a significant sign of CNS injury in COVID-19 patients (e.g., see previous works^102,103,135,136^), media reports of asymptomatic hypoxia suggest the need for early assessment of CNS involvement even in patients showing no neurological symptoms.

As has been the case for FDA-approved BBs of TBI,^217^ circulating biomarkers may prove useful to determine the need for emergency CTs, thereby assisting in allocation of limited medical resources during pandemics and mitigating the need to transport highly infectious patients to these imaging facilities. Both military and civilian healthcare emphasize the need for information on patients' abilities to return to normal daily routines. Thus, providing accurate prognoses of the extent and duration of neurological deficits in COVID-19 patients is important. As with TBI, sufficient clinical validation of BBs in CNS injury could contribute to aiding the clinical course of patients as well as their neurological outcomes.^220,221^

### Clinical applications for hospitalized patients and in intensive care units

A substantial percentage of COVID-19 patients are admitted for hospitalization or ICU care. Monitoring the status of possible CNS injury in these patients is vital given that cognitive deficits are observed even in ICU patients receiving care for other medical complications not associated with a primary brain injury.^199,205,241,332–334^ Even a year after leaving the ICU, many people experience PTSD, cognitive deficits, and depression.^335^ Thus, the presence or absence of CNS injury in the ICU will be important in determining subsequent neurological deficits and neuropsychiatric problems. ARDS in COVID-19 patients^336^ leads to ICU admission and is itself associated with long-term cognitive impairment.^337^

BBs also have clinical utility as diagnostics for detection of neurologic morbidity in ICU patients admitted for ARDS and multiple organ dysfunction syndrome,^338^ both typical manifestations of severe COVID-19. BBs could reduce need for neuroimaging as screening tests for potential CNS damage. In addition, sensitive and specific BBs could replace the option for diagnostic lumbar punctures to collect samples for assessments of possible CNS involvement. Appropriate use of clinically validated brain injury biomarkers could more safely, rapidly, and inexpensively inform clinicians on any ongoing, potentially evolving brain pathology, thereby guiding management and intervention strategies.

## Research Applications of Blood Biomarkers to Enhance Potential Utility of Blood Biomarkers for Risk Analyses and Clinical Trial Designs

### Assessments of risks for neurodegenerative diseases

**FIG. 1.**
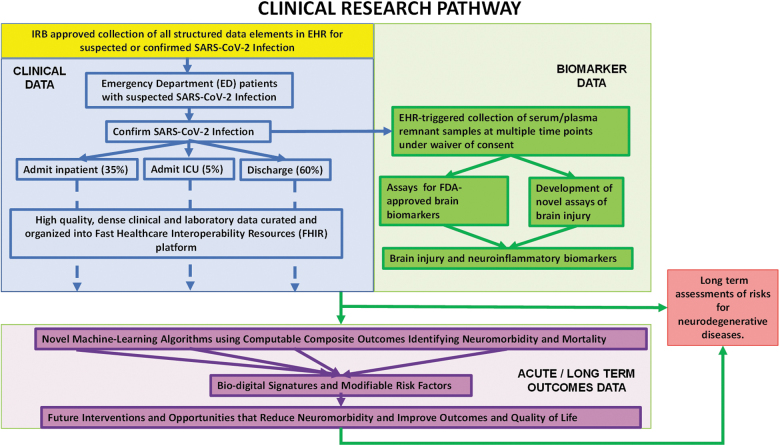
Generalized schematic of a clinical research study of BBs of CNS Injury in COVID-19 patients. BBs, blood biomarkers; CNS, central nervous system; COVID-19, coronavirus disease 2019; EHR, electronic health record; FDA, U.S. Food and Drug Administration; ICU, intensive care unit; IRB, institutional review board.

The assessment of possible risk factors for AD or other neurodegenerative diseases conferred by SARS-CoV-2 will require a long-term longitudinal study. Given the magnitude of the pandemic, an approach similar to the Framingham Heart Study could be warranted. Such a study should minimally include assessments of biomarkers already implicated in the early of stages of AD such as tau, beta amyloid, NfL, and GFAP^339,340^ (Fig. 1).

### Design of clinical trials of therapies to treat central nervous system injury

There is widespread recognition that the use of BBs, alone and in combination with other diagnostic tools, can improve design of therapeutic trials for acute CNS injuries and neurodegenerative diseases.^341,342^ BBs could also enhance the design of treatment trials designed to assess effectiveness of therapies for CNS injury in COVID-19 patients. A secondary analysis of BBs collected from moderate and severe TBI patients showed that BBs, especially GFAP, could reliably predict intracranial hemorrhages and outcomes.^342^ In addition to approving UCH-L1 and GFAP for acute diagnosis of mild versus moderate TBI, the FDA has issued a Letter of Support for the incorporation of these two BBs into clinical trials studying potential therapies for mild TBI.^218^ Operation Brain Trauma Therapy (OBTT) provided strong support for the use of BBs for pre-clinical drug development studies for TBI (e.g., see an earlier work^343^) and reflects increasing confidence in the use of BBs to detect and monitor CNS injury.

## Designing Clinical Studies to Examine the Utility of Blood Biomarkers for Diagnosis of Central Nervous System Injury: An Overview of a Clinical Research Strategy

Figure 1 presents a general outline of studies of BBs to diagnose CNS injury in COVID-19 patients. BBs data can be integrated with patient data and existing diagnostic technologies (brain imaging, neurological assessments, and genotyping) and readily incorporated into current standard-of-care practices. The pathway proposes serial collection of remnant blood samples acutely (admission to 72 h) under resource-limited pandemic conditions, daily (or more frequent) interrogation of BBs in hospitalized patients, and integration of BBs data with EHR-embedded records of neuromorbidity^344^ and other structured CDEs. In addition, neurological condition at discharge and long-term follow-up of patients (e.g., 6–12 months) should be integrated with the EHR data. Selection and frequency of sampling of BBs can be modified to meet the needs of individual clinical study designs. Selection of BBs should be carefully designed and specifically selected on the basis of the goals of the study. For example, living systematic reviews of BBs of TBI can provide updated resources (e.g., see a previous work^345^), and recent reviews of stroke^198^ can provide updated resources for BB review and selection.

There are a number of currently well-characterized BBs of brain injury that can be incorporated in studies of COVID-19.^198,203^ BBs should include GFAP, UCH-L1, S100B, and NfL, proteins extensively described in studies of acute brain injury.^198,202,203^ Sensitive and FDA-approved assays are available.^217^ In light of the potential importance of immunosuppression, inflammation, and “cytokine storms,”^143,144^ a systematic assessment of markers of inflammation should also be included into BBs selected for further study.^346^ It will be important to appropriately adjust sampling time points to identify potentially differing temporal profiles of individual biomarkers, a property which could assist in characterizing acute versus chronic pathological processes. Serial neurological assessments can be similarly adjusted. The pathway also provides for use of HIT to support integration of BBs data with other clinical and diagnostic information.

It is important that the designs of clinical research studies of BBs of CNS injury in COVID-19 patients generate data to assess the potential clinical utility of the BBs as outlined in the preceding section. Research approaches must also consider general clinical and pathobiological phenotypes resulting from SARS-CoV-2 CNS infection. These include: 1) the potential for SARS-CoV-2 to directly infect the brain producing acute CNS injury and subsequent persistent neurological deficits versus brain injury resulting from secondary systemic injuries; 2) the potential for previous brain injuries or existing neurodegenerative diseases to be associated with increased risks for and/or increased magnitudes of CNS injury; 3) the potential for hospitalized COVID-19 patients to have increased pathological responses to secondary systemic insults as a resulting of CNS infection by SARS-CoV-2; and 4) the potential for increased risk of subsequent neurodegenerative diseases such as AD or PD. It is unlikely that a single BB of brain injury will optimally differentiate the potential subsequent evolution into these phenotypes.

It is also essential that any study evaluating the utility of BBs review and report the analytical properties of the assay platform employed in the research (e.g., limits of quantification, lower limits of detection). Technological improvements have led to increased accuracy of the assays. Failure to provide the methodology and performance characteristics of BB assays makes it impossible to compare data from different studies and can lead to erroneous interpretations.^346^

## Conclusions

We are at the beginning of our understanding of the pathobiology of CNS infection by the SARS-CoV-2 virus and of the neurological consequences of this infection. The COVID-19 pandemic poses significant risks for acute and persistent neurological deficits, as well as possible increased risk for neurodegenerative diseases. The use of BBs of brain injury integrated with additional existing diagnostic tools with big dataset analytics could provide timely, cost-effective approaches to address this increasingly urgent unmet medical need.
